# A Glutathione-Nrf2-Thioredoxin Cross-Talk Ensures Keratinocyte Survival and Efficient Wound Repair

**DOI:** 10.1371/journal.pgen.1005800

**Published:** 2016-01-25

**Authors:** Michèle Telorack, Michael Meyer, Irina Ingold, Marcus Conrad, Wilhelm Bloch, Sabine Werner

**Affiliations:** 1 Department of Biology, Institute of Molecular Health Sciences, ETH Zurich, Zurich, Switzerland; 2 Helmholtz Center Munich, Institute of Developmental Genetics, Neuherberg, Germany; 3 Department of Molecular and Cellular Sport Medicine, German Sport University Cologne, Cologne, Germany; National Cancer Institute, National Institutes of Health, UNITED STATES

## Abstract

The tripeptide glutathione is the most abundant cellular antioxidant with high medical relevance, and it is also required as a co-factor for various enzymes involved in the detoxification of reactive oxygen species and toxic compounds. However, its cell-type specific functions and its interaction with other cytoprotective molecules are largely unknown. Using a combination of mouse genetics, functional cell biology and pharmacology, we unraveled the function of glutathione in keratinocytes and its cross-talk with other antioxidant defense systems. Mice with keratinocyte-specific deficiency in glutamate cysteine ligase, which catalyzes the rate-limiting step in glutathione biosynthesis, showed a strong reduction in keratinocyte viability *in vitro* and in the skin *in vivo*. The cells died predominantly by apoptosis, but also showed features of ferroptosis and necroptosis. The increased cell death was associated with increased levels of reactive oxygen and nitrogen species, which caused DNA and mitochondrial damage. However, epidermal architecture, and even healing of excisional skin wounds were only mildly affected in the mutant mice. The cytoprotective transcription factor Nrf2 was strongly activated in glutathione-deficient keratinocytes, but additional loss of Nrf2 did not aggravate the phenotype, demonstrating that the cytoprotective effect of Nrf2 is glutathione dependent. However, we show that deficiency in glutathione biosynthesis is efficiently compensated in keratinocytes by the cysteine/cystine and thioredoxin systems. Therefore, our study highlights a remarkable antioxidant capacity of the epidermis that ensures skin integrity and efficient wound healing.

## Author Summary

The skin is frequently exposed to various challenges that cause oxidative stress, such as UV irradiation, exposure to toxic chemicals or mechanical injury. Therefore, keratinocytes -the major cell type of the outermost layer of our skin—need sophisticated antioxidant defense systems. However, the relevance of individual defense systems and their interaction in the cell are largely unknown. Here we studied the role of glutathione, the most abundant cellular antioxidant, in keratinocytes. We show that in the absence of the enzyme that catalyzes the rate-limiting step in glutathione biosynthesis, keratinocytes initiate a complex cell death program, resulting in reduced survival of these cells in culture and in mouse skin. Despite this, the epidermal structure was only mildly affected and the mutant mice were even able to heal full-thickness excisional skin wounds. We found that this is ensured by compensatory functions of another antioxidant defense system, allowing survival of keratinocytes in the absence of glutathione. These results therefore demonstrate a remarkable antioxidant defense capacity of keratinocytes that guarantees maintenance of the essential barrier of the skin and efficient wound repair.

## Introduction

Maintenance of the cellular redox balance is essential for appropriate cellular function. This balance is frequently challenged, particularly in response to infections or tissue damage inflicted by mechanical, chemical or physical insults. Under these conditions cells produce an excess of reactive oxygen and nitrogen species (ROS and RNS). While low levels of these molecules are required for cellular signaling [1], high levels can damage all types of cellular macromolecules [2]. Therefore, cells strongly depend on efficient ROS/RNS detoxification systems. This is achieved by various ROS/RNS-detoxifying enzymes, but also by low molecular weight antioxidants, such as vitamins C and E. Of particular importance is reduced glutathione (GSH), a tripeptide composed of glutamate, cysteine, and glycine. It is highly abundant in all cell types (≈ 1–10 mM), serves as a cellular redox buffer, and protects against the toxicity of electrophilic compounds. It also reacts with nitric oxide (NO) to yield nitrosoglutathione, thereby reducing the amount of free NO. Furthermore, it is used as a co-factor for glutathione peroxidases and by glutathione S-transferases for the glutathionylation of selected proteins and for conjugation of toxic substances, and it is required for the maturation of cytosolic iron-sulfur proteins [3–5]. Due to these vital functions, GSH deficiency plays a key role in the pathogenesis of major human diseases, including neurodegenerative diseases and diabetes, whereas increased GSH levels reduce the susceptibility of cancer cells to oxidative stress and therefore promote cancer progression and resistance to chemo- and radiotherapy [6, 7].

Since the skin forms the outermost barrier to the environment and is therefore frequently exposed to ultraviolet (UV) irradiation, toxic chemicals, or mechanical insults, the cytoprotective functions of GSH are likely to be of particular importance in this tissue. Indeed, esterified GSH prevented apoptosis of cultured keratinocytes under hyperglycemic conditions [8]. Upon UVA or UVB irradiation of cultured fibroblasts or keratinocytes, depletion of GSH occurred [9, 10], resulting in oxidative stress and cell death [11]. A role for GSH in wound repair has been suggested, since GSH depletion occurred in skin wounds, especially in situations of impaired healing, such as in immunocompromised rats, in diabetic mice and in human diabetic foot ulcers [12, 13]. Furthermore, GSH levels were reduced in wounds of aged compared to young rats [14], and chemical depletion of GSH in rat wounds reduced wound bursting strength [15]. On the other hand, topical treatment of poorly healing wounds in diabetic mice with esterified GSH accelerated the repair process [13]. Hence, all these studies strongly suggest that normal GSH levels are crucial for efficient wound healing.

So far GSH functions have mainly been studied with chemical agents that deplete the tripeptide, in particular buthionine sulfoximine (BSO). However, this approach does not allow long-term GSH depletion and analysis of cell-type specific activities *in vivo*. The generation of animals deficient in the rate-limiting enzyme in GSH biosynthesis—glutamate cysteine ligase (Gcl)—now allows addressing the role of GSH in tissue homeostasis, repair and disease. Gcl is a heterodimer consisting of a catalytic (Gclc) and a “regulatory” modifier (Gclm) subunit, and catalyzes the formation of γ-glutamylcysteine. Whereas Gclc possesses catalytic activity, Gclm cannot synthesize the dipeptide, but serves to change the kinetic characteristics of Gclc [4]. Mice lacking Gclm in all cells are viable and fertile, in spite of a strong reduction in GSH levels [16]. By contrast, *Gclc* knockout mice die *in utero* from enhanced apoptotic cell death [17, 18]. However, the affected cell types and the underlying mechanisms have not been characterized in detail.

Here, we analyzed the consequences of Gclc deficiency in keratinocytes, a cell type that continuously proliferates under steady state conditions and is frequently exposed to various external challenges. We demonstrate that GSH protects from DNA and mitochondrial damage and consequently ensures survival of keratinocytes in normal and wounded skin. Loss of GSH was partially compensated by the thioredoxin system, but not by activation of the cytoprotective transcription factor nuclear factor (erythroid-derived 2)-like 2 (Nrf2). Thus, keratinocytes have developed a remarkable combination of protective strategies that contribute to the efficient barrier function of the epidermis and help to maintain skin integrity even under stress conditions.

## Results

### Generation of mice lacking Gclc in keratinocytes

**Fig 1:**
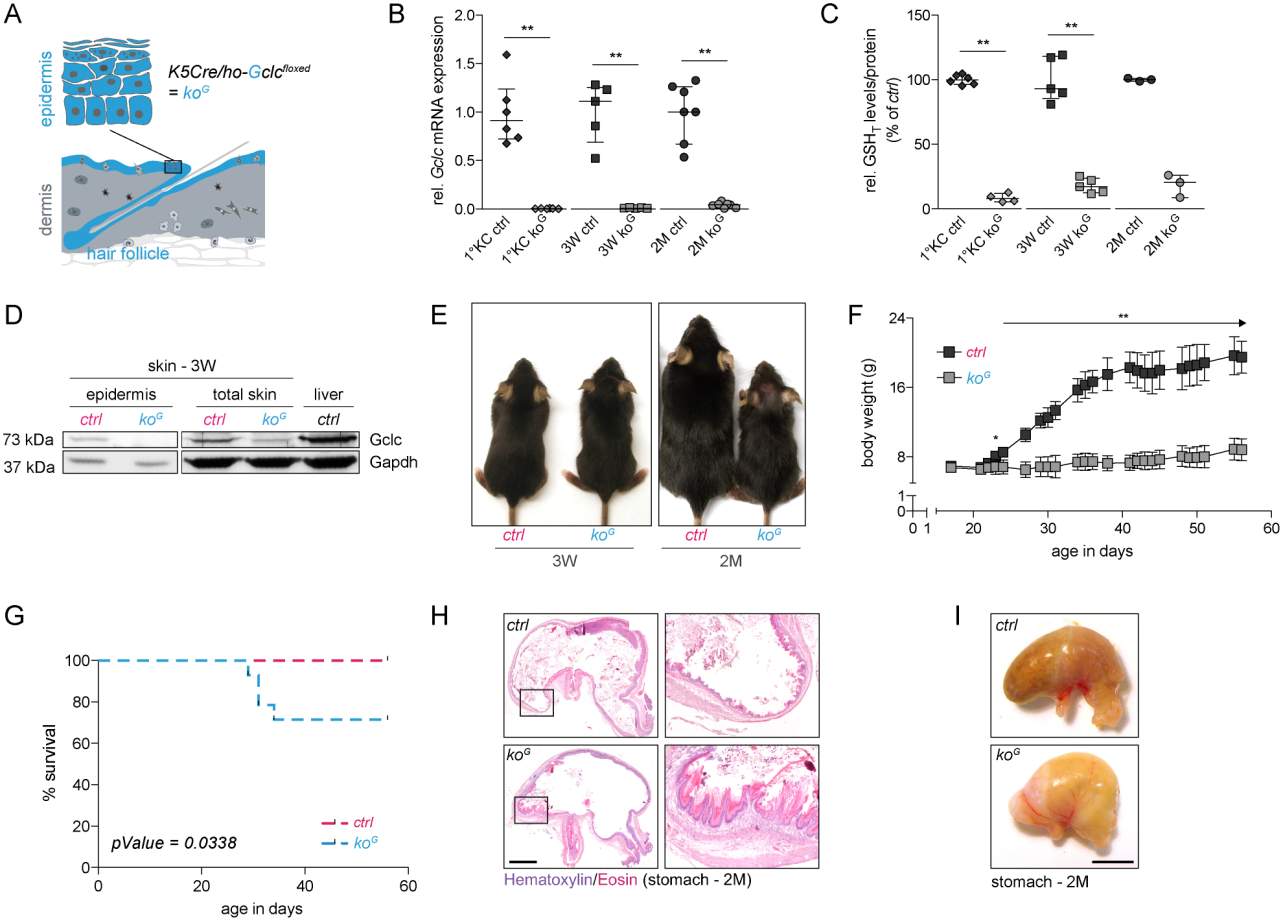
*Ko*^*G*^ mice are viable, but fail to gain weight after weaning. (A) Scheme of the skin. Keratinocytes of the epidermis and the hair follicles/sebaceous glands, which are affected by the knockout, are shown in blue. Mice heterozygous for the *K5Cre* transgene and homozygous for the floxed *Gclc* allele (ho-Gclc^floxed^) lack Gclc in keratinocytes (*ko*^*G*^ mice) (B) qRT-PCR of *Gclc* relative to *Rps29* using RNA from primary keratinocytes (1°KC; N = 6) or epidermis (3W N = 5; 2M N = 7). (C) Total GSH/GSSG levels (GSH_T_) in 1°KC (N = 6/4) and epidermis (3W N = 5; 2M N = 3). (D) Western blot with lysates from epidermis (pooled from 2 mice per genotype), total skin, and liver for Gclc and Gapdh (loading control). (E) Macroscopic appearance of *ko*^*G*^ and *ctrl* mice at 3W and 2M. (F) Body weight of *ctrl* and *ko*^*G*^ mice surviving until 2M. Arrow indicates **P ≤ 0.01 for each time point after P23. N = 7/5. (G) Kaplan-Meier survival curves of *ko*^*G*^ versus *ctrl* mice. N = 14. Significance was analyzed using Log-rank test. *P ≤ 0.05. (H) Hematoxylin/eosin (H&E) staining of stomach sections from mice at 2M. Scale bar: 1 mm. Squares indicate the area shown at higher magnification on the right side. Note the hyperkeratosis in the forestomach of *ko*^*G*^ mice. (I) Picture of the stomach from *ko*^*G*^ and *ctrl* mice at 2M. Scale bar: 0.5 cm. Scatter plots show the median with interquartile range.

We generated mice lacking Gclc in all keratinocytes (designated *ko*^*G*^ mice) by deleting the *Gclc* gene in the epidermal basal layer and in the outer root sheath keratinocytes of hair follicles using mice expressing Cre recombinase under control of the keratin 5 (K5) promoter (Fig 1A). The mutant mice were designated *ko*^*G*^ mice. Loss of *Gclc* expression was verified by qRT-PCR using epidermal RNA from mice at the age of 3 weeks (3W) or two months (2M) and from cultured primary keratinocytes (1°KC—Fig 1B). The residual levels of *Gclc* transcripts in the epidermis most likely result from expression in epidermal cells other than keratinocytes, which are not targeted by the keratin 5 promoter. A similar result was obtained for total GSH/GSSG levels (Fig 1C). Loss of Gclc protein in the epidermis and reduced Gclc levels in total skin were confirmed by Western blot analysis (Fig 1D). Since Gclc expression was still almost undetectable at 2M, it seems unlikely that cells, which had escaped recombination, predominantly contribute to epidermal regeneration. The loss of Gclc in keratinocytes did not affect the expression of the glutathione-dependent enzymes peroxiredoxin 6 (Prdx6) and glutathione peroxidase 4 (Gpx4), while expression levels of glutaredoxin 2 (Glrx2) were slightly increased (S1A and S1B Fig).

### Progressive phenotypic abnormalities in Gclc-deficient mice

At 3W of age Gclc-deficient mice were first distinguishable from littermate controls by the rough appearance of their fur (Fig 1E). Patchy hair loss was observed in some knockout mice (Fig 1E right panel; see head region, and S1C Fig) and the skin appeared less elastic (S1D Fig). In addition, the hairs of *ko*^*G*^ mice were thinner and showed malformation (S1E Fig). The hair loss was not due to loss of hair follicles, which showed the same or even a mildly increased density in *ko*^*G*^ mice (S1F Fig). Rather, it is most likely the consequence of severe hyperkeratosis in the hair follicle infundibulum (S1G Fig), which affects the anchorage of the hairs and may also cause the observed hair malformation due to narrowing of the hair canal.

*Ko*^*G*^ mice failed to gain weight after weaning (Fig 1F) and approximately 25% of them died or had to be sacrificed according to animal welfare regulations because of their general weakness (Fig 1G). The lack of weight gain was most likely due to malnutrition that resulted from hyperkeratosis in the forestomach (Fig 1H), where the K5 promoter is also active [19]. Stomach abnormalities were also reflected by the severe bloating of the stomach (Fig 1I). Blood glucose levels of *ko*^*G*^ mice were significantly lower compared to control mice at 3W and 2M (S2A Fig), further suggesting malnutrition as the cause of cachexia. By contrast, it is not the consequence of tooth abnormalities (S2B Fig) or of abnormal activity of the K5 promoter in tissues lacking K5 expression, such as the liver or the kidney (S2C Fig), which would result in GSH deficiency in other organs relevant for whole body metabolism. Furthermore, systemic inflammation as a reason for the lack of weight gain seems unlikely, since serum levels of the pro-inflammatory cytokine interleukin-6 (Il-6), a marker for systemic inflammation, were below the detection levels of the ELISA (0.05 ng/ml).

### Gclc-deficient mice develop hyperkeratosis

**Fig 2:**
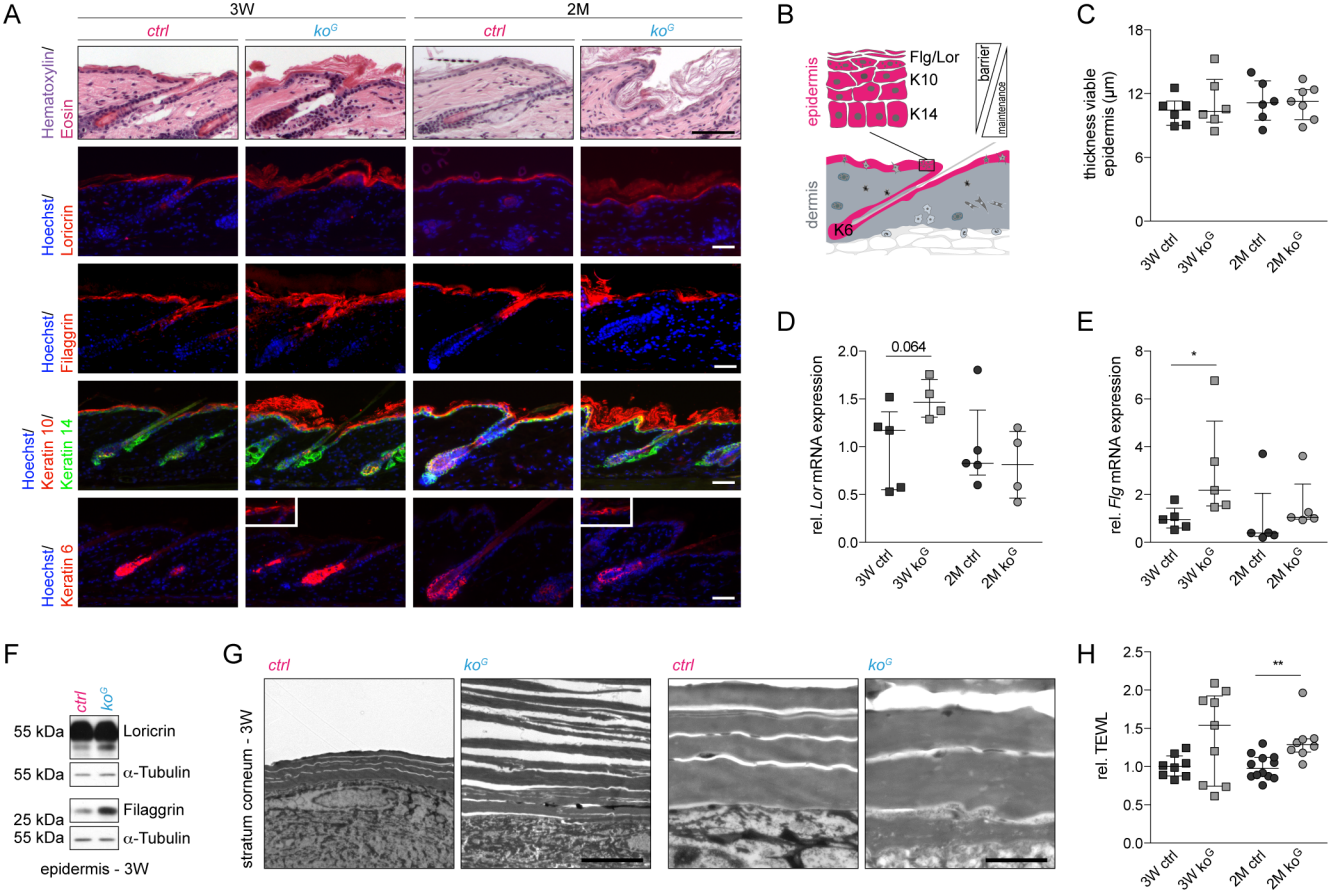
Progressive skin abnormalities in *ko*^*G*^ mice. (A) H&E staining of longitudinal skin sections from mice at 3W and 2M and immunofluorescence staining for epidermal differentiation markers. Smaller pictures within the *ko*^*G*^ panel show patches of interfollicular K6. Nuclei were counterstained with Hoechst. Scale bars: 200 μm (H&E) or 40 μm (immunofluorescence). Note the scaling in *ko*^*G*^ mice. The appropriate expression pattern of the differentiation markers and their function in the epidermis are shown schematically in (B). K = Keratin, Flg = Filaggrin, Lor = Loricrin. (C) Thickness of the viable epidermis (all layers except the stratum corneum). 3W (N = 6) and 2M (N = 6/7). (D,E) qRT-PCR of *Lor* (N = 5/4) and *Flg* (N = 5) relative to *Rps29* using RNA from the epidermis at 3W and 2M. (F) Western blot of epidermal lysates at 3W for Lor, Flg and *α*-Tubulin (loading control). Shown is one representative sample for each genotype (N = 4). (G) Electron microscopy of the *stratum corneum* at 3W showing reduced adhesiveness of the corneocytes in the epidermis of *ko*^*G*^ mice. Scale bars: 5.5 μm (left panels) or 2.5 μm (right panels). (H) TEWL of mice at 3W (N = 8/9) and 2M (N = 12/8). Scatter plots show the median with interquartile range. *P ≤ 0.05, **P ≤ 0.01.

Cutaneous abnormalities were not detected prior to P18 (S3A Fig). From 3W onwards, progressive hyperkeratosis was observed as reflected by hematoxylin/eosin (H&E) staining and immunofluorescence analysis of the late differentiation marker loricrin, which had a broader and more diffuse distribution in the knockout mice (Fig 2A). Furthermore, a more intense staining for filaggrin, a structural protein of the stratum corneum (Fig 2B), was observed in the 3W *ko*^*G*^ epidermis, followed by patchy expression of filaggrin in the mutant mice at 2M (Fig 2A). The thickness of the viable epidermis was not affected by the loss of Gclc (Fig 2C), and keratin 14 (K14) and K10 were appropriately expressed in the basal or suprabasal layers, respectively (Fig 2A and 2B). However, the K10 positive area was extended (Fig 2A). In addition, some patches of interfollicular K6 positive areas were detected (Fig 2A), demonstrating mild abnormalities in keratinocyte differentiation. qRT-PCR and Western blot analysis of epidermal RNAs/lysates confirmed the mild increase in loricrin expression and the stronger increase in filaggrin expression in young mice (Fig 2D–2F). Processing of filaggrin was not affected (S3B Fig). Hyperkeratosis in *ko*^*G*^ mice was also detected by electron microscopy, which revealed reduced attachment of the corneocytes (Fig 2G). These *stratum corneum* abnormalities correlate with a mild increase in transepidermal water loss (TEWL), which reflects a defect in the epidermal barrier (Fig 2H). This was already seen in some mice at the age of 3W, and the difference was statistically significant at the age of 2M.

### Keratinocyte hyperproliferation and mild skin inflammation in Gclc-deficient mice

**Fig 3:**
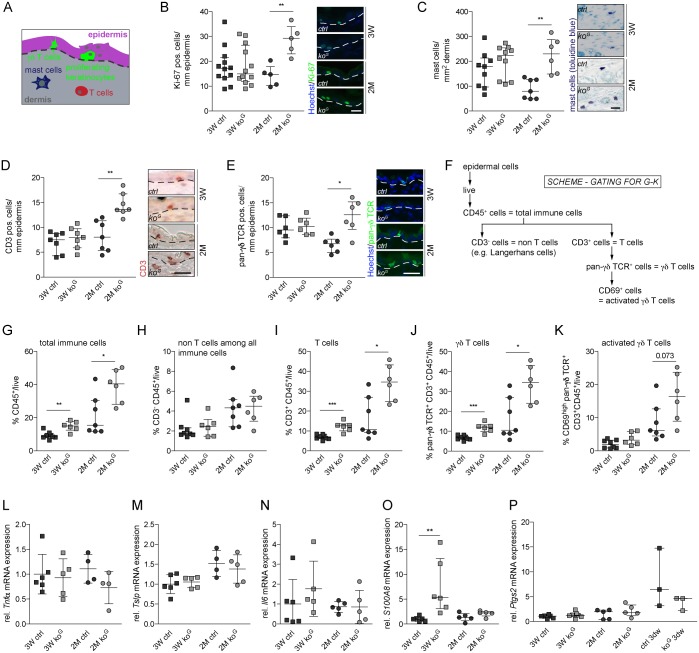
Keratinocyte hyperproliferation and mild inflammation in the skin of *ko*^*G*^ mice. (A) Scheme of the skin visualizing the region analyzed in (B-E) and the different cell types of the skin that were analyzed in this study. (B) Immunofluorescence staining of skin sections for Ki-67 and quantification of positive cells per length epidermis (3W N = 12; 2M N = 5). Scale bar: 20 μm. (C) Toluidine blue staining of mast cells and quantification of positive cells per area dermis (3W N = 9/10; 2M N = 7/6). Scale bar: 10 μm. (D,E) Immunohistochemistry of the T cell marker CD3 (3W N = 7/7; 2M N = 7) and immunofluorescence for the γδ T cell receptor (3W/2M N = 6). The number of positive cells per length epidermis was determined. Scale bar: 10 μm. White/black dotted lines in (B,D,E) indicate the basement membrane. (F) Schematic representation of the gating strategy used for the detection of epidermal immune cells by flow cytometry. (G-K) Flow cytometry analysis of epidermal cells from mice at 3W or 2M using markers for different types of immune cells and their activation status (3W N = 8/6; 2M N = 7/6). (L-P) qRT-PCR of *Tnfα*, *Tslp*, *Il6*, *S100A8* and *Ptgs2* (N = 3–6) relative to *Rps29* using RNA from the epidermis at 3W and 2M. For *Ptgs2*, which was hardly detectable in the normal epidermis, epidermis from wounded skin (3 days after wounding) from ctrl and *ko*^*G*^ mice was used as positive control. Scatter plots show the median with interquartile range. *P ≤ 0.05, **P ≤ 0.01, ***P ≤ 0.001.

The rate of keratinocyte proliferation was not reduced in *ko*^*G*^ mice at 3W. On the contrary, keratinocytes became hyperproliferative at the age of 2M (Fig 3A and 3B). The late onset suggests that keratinocyte hyperproliferation is a non-cell autonomous effect, and may result from an increase in cutaneous immune cells, which are known producers of keratinocyte mitogens [20]. Consistent with this assumption, toluidine blue staining revealed an increase in the number of dermal mast cells, and immunofluorescence staining showed that the number of epidermal γδ T cells was significantly increased at 2M (Fig 3C–3E). A more detailed analysis of the epidermal immune cells by flow cytometry confirmed the increase in epidermal γδ T cells, although the number of γδ T cells expressing the activation marker CD69 was not significantly increased (Figs 3F–3K and S4A). This accumulation started at the age of 3W and became more obvious at 2M. Analysis of dermal cells by flow cytometry showed no increase in immune cells in *ko*^*G*^ mice at the age of 3W, a significant increase in the number of all immune cells at the age of 2 months, but no differences in the number of macrophages and neutrophils at any time point (S4B and S4C Fig).

The mild inflammation, however, was not associated with a significant increase in the expression of the pro-inflammatory cytokines tumor necrosis factor alpha (*Tnfα*) and *Il6*, and of thymic stromal lymphopoietin (*Tslp*) in the skin of *ko*^*G*^ mice (Fig 3L–3N). Expression of *S100A8* was increased in the epidermis of young *ko*^*G*^ mice, but returned to basal levels at the age of 2M (Fig 3O). Finally, there was no increase in the expression of prostaglandin-endoperoxide synthase 2 (*Ptgs2*, also known as cyclooxygenase-2), suggesting that prostaglandin production is not enhanced (Fig 3P). These findings demonstrate that there is only a mild cutaneous inflammation in *ko*^*G*^ mice and that the lack of weight gain is most likely not a consequence of systemic abnormalities that result from enhanced production and release of pro-inflammatory cytokines in the skin.

### Mild wound healing abnormalities in *ko*^*G*^ mice

**Fig 4:**
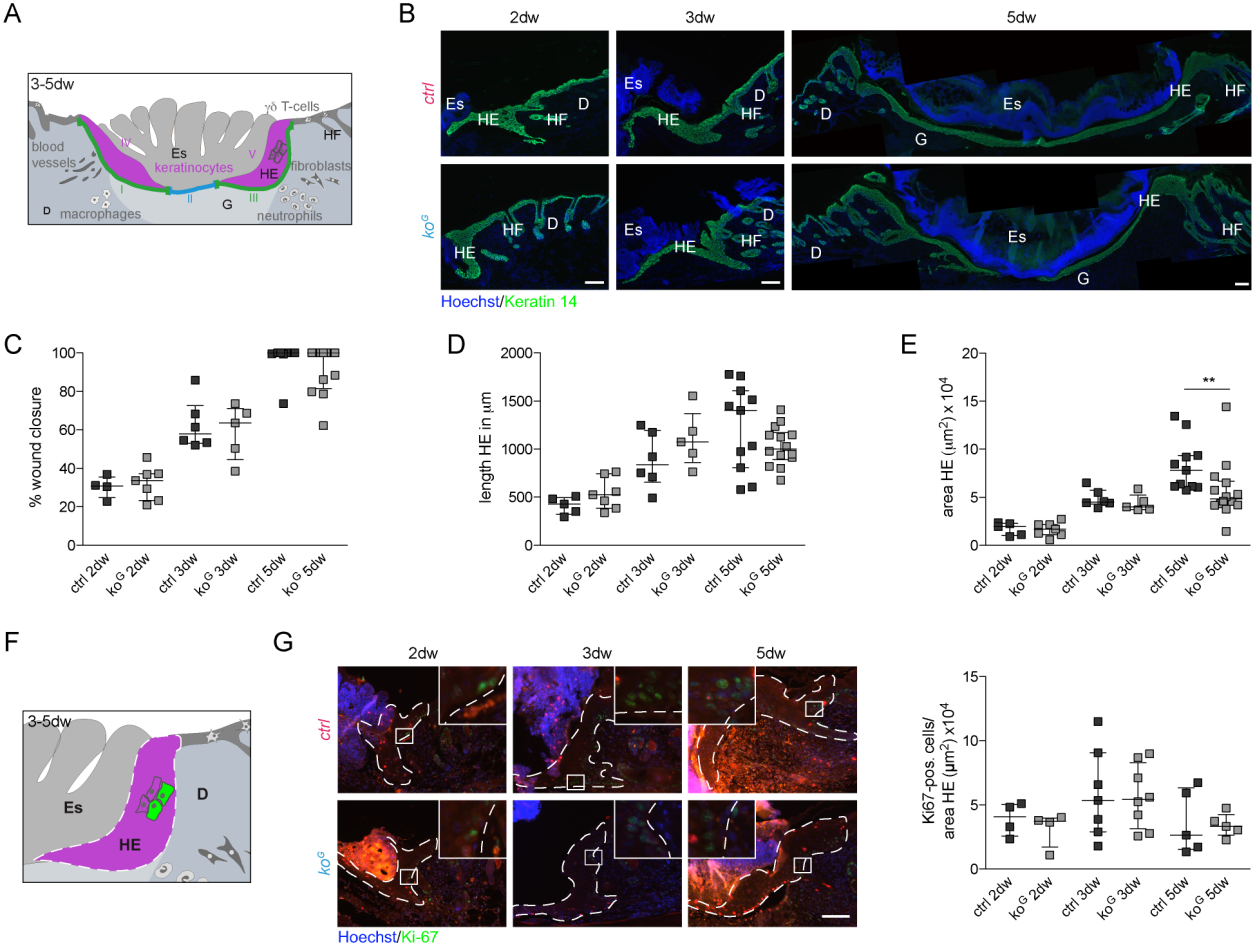
Mild wound healing abnormalities in *ko*^*G*^ mice. (A) Schematic representation of a 3–5 day wound showing the parameters for morphometric analysis: percentage of wound closure = (I+III/I+II+III)*100; length HE = average length I+III; area HE = average area IV+V. D: dermis Es: eschar, G: granulation tissue, HF: hair follicle, HE: hyperproliferative epithelium. (B) Immunofluorescence staining of K14 (green) and Hoechst counterstaining (blue) on sections from 3- and 5-day wounds. Scale bar: 100 μm. (C-E) Morphometric analysis of (C) percentage of wound closure, (D) length HE, and (E) area HE of 2-, 3- and 5-day wounds. For (C): 2dw N = 4/7; 3dw N = 6/5; 5dw N = 9/12. For (D): 2dw N = 5/7; 3dw N = 6/5; 5dw N = 11/14. For (E): 2dw N = 5/7; 3dw N = 6/5; 5dw N = 11/14. (F) Schematic representation of the HE with the region of the cells that were analyzed in (G). (G) Immunofluorescence-stained sections of 2-, 3- and 5-day wounds were used for quantification of Ki-67 positive cells per area HE. White dotted lines show the area of HE in which positive cells were counted. Each picture includes a magnification of the indicated square to show positive cells. Scale bar = 100 μm. 2dw N = 4; 3dw N = 7/8; 5dw N = 5. Scatter plots show the median with interquartile range. **P ≤ 0.01.

Since chemical Gcl inhibitors strongly affected the healing of rat incisional wounds [15], we analyzed the healing of full-thickness excisional wounds in *ko*^*G*^ and control mice. These experiments were performed prior to the development of health deficits and severe skin abnormalities (P19-24) to exclude that any healing abnormalities are secondary to the malnutrition or to pre-existing histological abnormalities in the skin. Histomorphometric analysis of stained wound sections using the parameters shown in Fig 4A revealed no difference in wound closure and length of the migrating epithelium at days 2, 3 and 5 after wounding (Fig 4B–4D). There was a mild, but significant reduction in the area of the wound epithelium in *ko*^*G*^ mice compared to controls at day 5 after wounding (Fig 4E), which, however, did not result from reduced keratinocyte proliferation (Fig 4F and 4G).

### Activation of Nrf2 does not compensate for the loss of Gclc in keratinocytes

**Fig 5:**
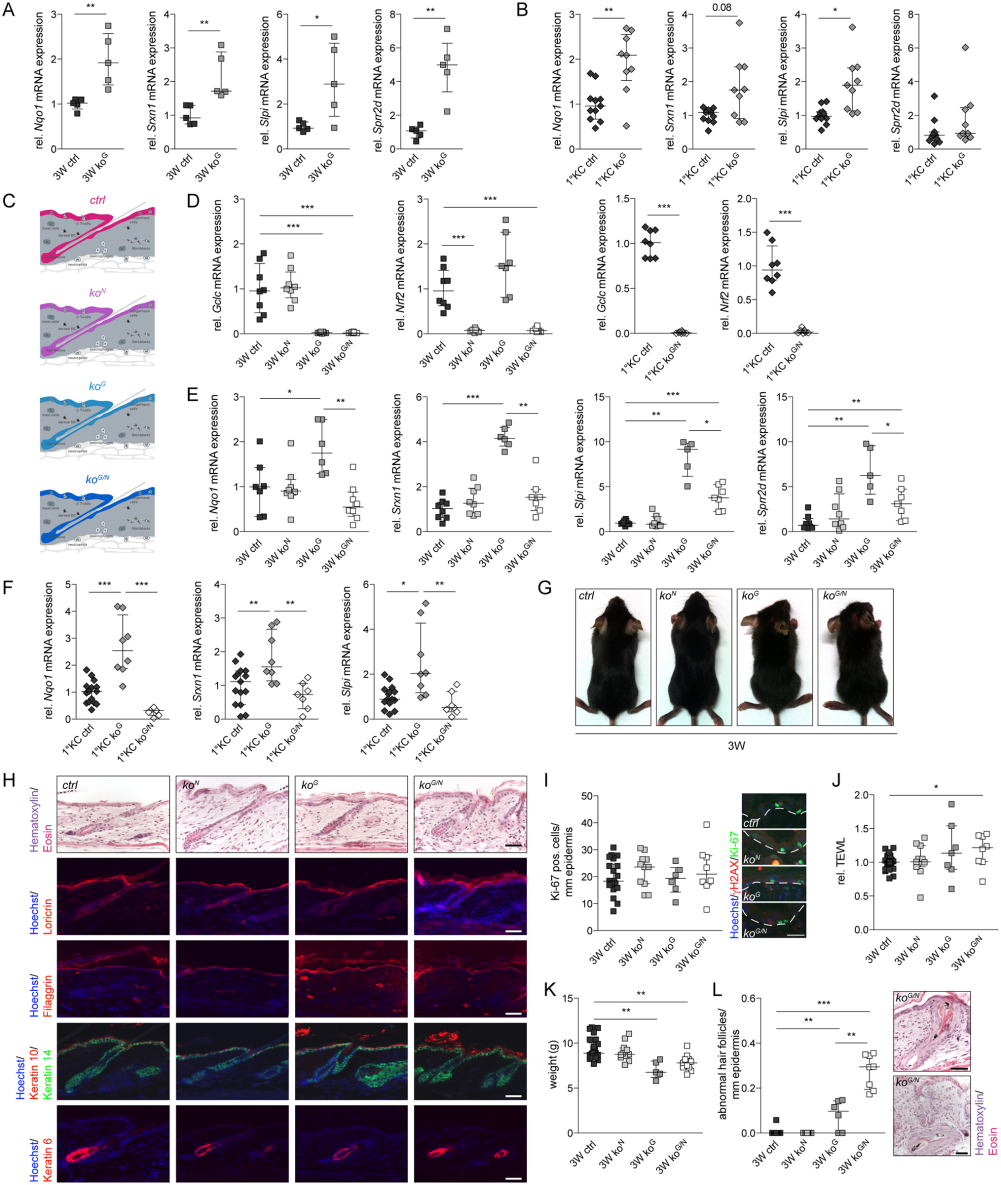
Nrf2 is activated in *ko*^*G*^ mice, but does not compensate for the loss of Gclc. (A,B) qRT-PCR of *Nqo1*, *Srxn1*, *Slpi* and *Sprr2d* relative to *Rps29* using RNA from (A) the epidermis at 3W (N = 5) and from (B) primary keratinocytes (N = 11/9). (C) Scheme of the skin from mice of all four genotypes used for the analysis. Keratinocytes affected by the knockout are shown in the respective color. (D) qRT-PCR of *Gclc* and *Nrf2* relative to *Rps29* using RNA from epidermis at 3W (left panel; N = 8/8/7/8) or primary keratinocytes of control and *ko*^*G/N*^ mice (right panel; N = 8). (E,F) qRT-PCR of *Nqo1*, *Srxn1*, *Slpi*, and *Sprr2d* relative to *Rps29* using RNA from the epidermis at 3W (N = 7-8/7-8/5-6/7-8) and of *Nqo1*, *Srxn1*, *Slpi* relative to *Rps29* using RNA from primary keratinocytes (N = 15/8/7). (G) Macroscopic appearance of mice of all four genotypes at 3W. (H) H&E staining of longitudinal skin sections from mice at 3W and immunofluorescence staining for epidermal differentiation markers. Nuclei were counterstained with Hoechst. Scale bars: 40 μm. (I) Immunofluorescence staining of skin sections for Ki-67 and quantification of positive cells per length epidermis at 3W (N = 22/11/6/8). Scale bar: 20 μm. White dotted lines indicate the basement membrane. (J) TEWL of mice at 3W (N = 25/11/7/8). (K) Body weight of mice at 3W (N = 26/11/5/11). (L) H&E staining of skin from *ko*^*G/N*^ mice at 3W demonstrating malformed hair follicles and cyst formation. The number of abnormal hair follicles per length epidermis is shown in the bar graph (N = 9/9/6/8; n.d. = not detectable). Scale bar: 40 μm. Scatter plots show the median with interquartile range. *P ≤ 0.05, **P ≤ 0.01, ***P ≤ 0.001.

The unexpectedly weak epidermal phenotype of Gclc-deficient mice, even in response to wounding, suggested that other proteins or low molecular weight antioxidants might compensate at least in part for the loss of Gclc in keratinocytes. A likely candidate is the Nrf2 transcription factor, which controls the expression of a battery of cytoprotective genes [21] and which was shown to be activated upon chemical GSH depletion [22, 23]. Furthermore, activation of Nrf2 in the skin either pharmacologically or through expression of a constitutively active Nrf2 mutant (caNrf2) in keratinocytes of transgenic mice caused acanthosis and hyperkeratosis in the epidermis and in the hair follicle infundibuli combined with patchy hair loss—a similar phenotype as seen in *ko*^*G*^ mice [24]. Indeed, a strong increase in the expression of the well-established Nrf2 target genes NAD(P)H dehydrogenase quinone 1 (*Nqo1)* and sulfiredoxin-1 (*Srxn1*) was observed in the epidermis of *ko*^*G*^ mice at 3W and 2M, together with upregulation of secretory leukocyte peptidase inhibitor (*Slpi*) and small proline-rich protein 2D (Figs 5A and S5A). The overexpression of these recently identified Nrf2 target genes had been shown to be responsible for the epidermal and the hair follicle phenotype of mice expressing caNrf2 in keratinocytes [24, 25]. Upregulation was confirmed for additional Nrf2 target genes at 3W (S5B Fig) and for the gene encoding “Elongation of very long chain fatty acids protein 3” (Elovl3) (S5C Fig). This enzyme is involved in the production of long chain fatty acids, and it is indirectly regulated by caNrf2 in keratinocytes [25]. Its abnormal expression may contribute to the barrier function defect of caNrf2-transgenic and of *ko*^*G*^ mice due to alterations in the lipid barrier. Other Elovls, however, where not dysregulated in the *Gclc* knockout epidermis (S5C Fig), which might explain the milder barrier defect of *ko*^*G*^ mice compared to caNrf2-transgenic mice. Activation of Nrf2 in the absence of Gclc is a cell autonomous effect, since expression of Nrf2 target genes was also upregulated in cultured Gclc-deficient keratinocytes (Fig 5B). By contrast, expression of superoxide dismutase 1 and catalase was neither affected *in vitro* nor *in vivo* (S5D Fig). Furthermore, expression of L-gulonolactonoxidase, an enzyme essential for the production of the antioxidant ascorbate (Vitamin C), could not be detected in epidermal and total skin samples of ctrl and *ko*^*G*^ mice, whereas it was readily detectable in liver samples (S5D Fig).

To determine if the activation of Nrf2 has a compensatory function, we generated mice lacking both Gclc and Nrf2 in keratinocytes (designated *ko*^*G/N*^ mice) (Fig 5C and 5D). The enhanced expression of Nrf2 target genes that occurred in Gclc-deficient epidermis was indeed completely (*Nqo1* and *Srxn1*) or partially (*Slpi* and *Sprr2d*) rescued (Fig 5E). This was confirmed for some of the genes in primary keratinocytes (Fig 5F). In contrast to our expectations, *ko*^*G/N*^ mice did not exhibit a more severe macroscopic or histological phenotype compared to *ko*^*G*^ mice at 3W (Fig 5G and 5H), and the expression of keratinocyte differentiation markers was also not obviously affected by the loss of Nrf2 (Fig 5H). There was also no reduction of the hyperkeratosis, and the squames were even more detached (Fig 5H). The additional loss of Nrf2 had also no effect on keratinocyte proliferation, TEWL, and body weight (Fig 5I–5K). The only additional abnormalities were misalignment and malformation of hair follicles combined with cyst formation (Fig 5H and 5L). Even at 2M of age, when the phenotype in single *Gclc* knockout mice becomes more severe, we did not observe additional abnormalities in skin morphology, body weight or TEWL in *ko*^*G/N*^ mice (S5E–S5G Fig).

### Gclc deficiency causes keratinocyte apoptosis and DNA damage

**Fig 6:**
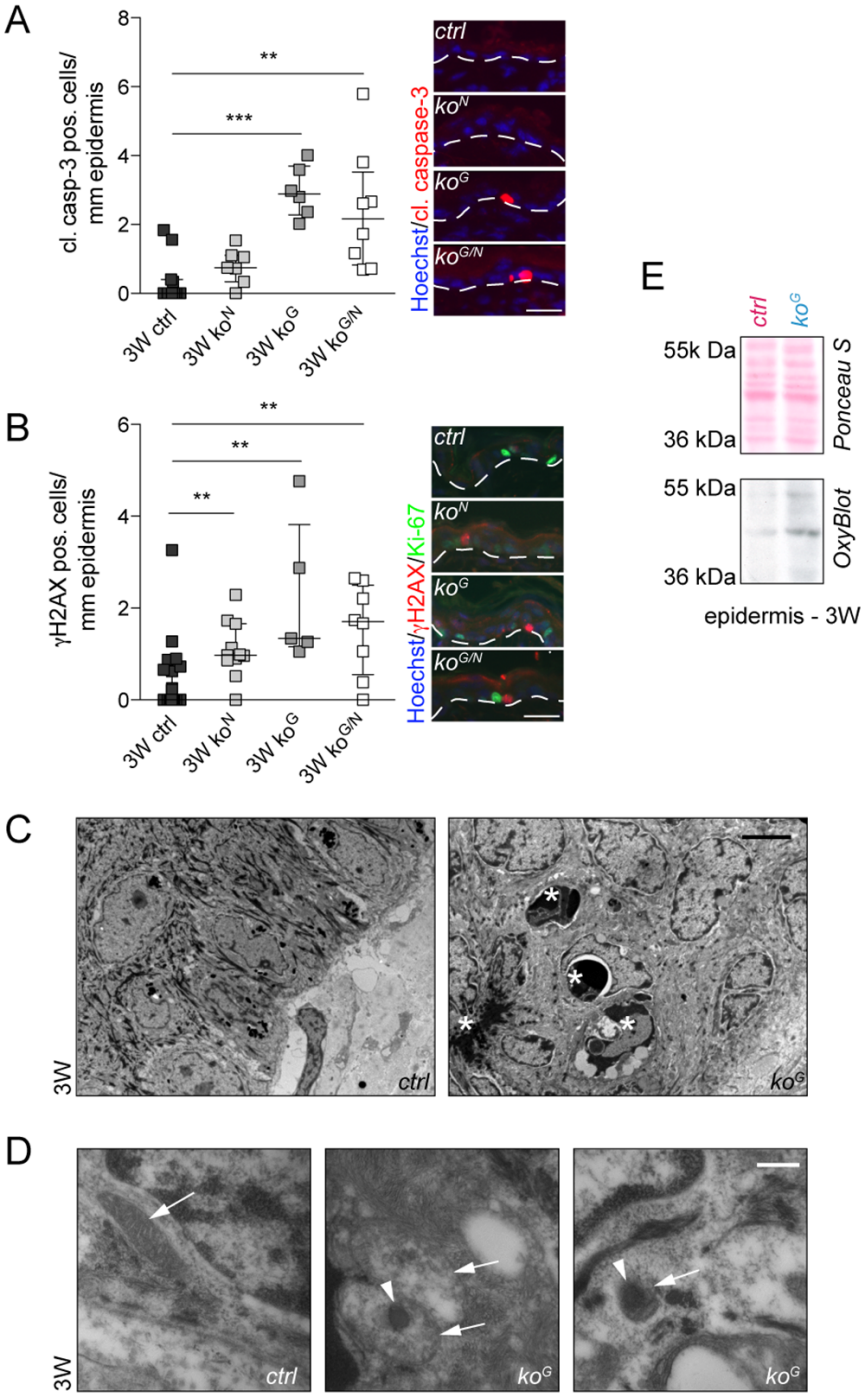
Enhanced apoptosis and DNA damage in the epidermis of Gclc-deficient mice. (A,B) Immunofluorescence staining of skin sections at 3W for cleaved caspase-3 (N = 11/7/6/8) and γH2AX (N = 22/11/5/8) and quantification of positive cells per length epidermis. White dotted lines indicate the basement membrane. Scale bar: 20 μm. Scatter plots show the median with interquartile range. **P ≤ 0.01, ***P ≤ 0.001. (C,D) Electron microscopy of epidermis at 3W shows dermal-epidermal border with the basal and suprabasal layers of the epidermis. (C) Apoptotic keratinocytes in the basal and suprabasal layers have condensed nuclei separated by a small cleft from the cytosol (asterisk). Scale bar: 3 μm. (D) Basal keratinocytes from control mice show a well-organized mitochondrium (arrow) with well recognizable and lamellar organized cristae (left panel). The matrix of mitochondria in apoptotic (middle panel) and non-apoptotic (right panel) basal keratinocytes of *ko*^*G*^ mice lacks the typical cristae structure and reveals only small short cristae in disorganized orientation. Furthermore, the damaged mitochondrial matrix shows dense agglomeration (arrowheads). Scale bar: 170 nm. (E) OxyBlot of epidermal tissue lysates at 3W. Equal loading was confirmed by Ponceau S staining of the membrane. Shown is one representative sample for each genotype (N = 5).

We next determined if the epidermal abnormalities in *ko*^*G*^ mice and the reduced area of the wound epidermis result from enhanced cell death. Indeed, a significant increase in the number of cleaved caspase-3 positive (apoptotic) keratinocytes was observed in the epidermis of *ko*^*G*^ mice with an early onset already at P2.5 and prior to the development of macroscopic or histological abnormalities (Figs 6A and S6A), but there was no further increase by concomitant loss of Nrf2 (Fig 6A). Consistent with the initiation of apoptosis by stress-induced activation/stabilization of p53 [26], more epidermal keratinocytes were positive for nuclear p53 in the absence of Gclc, while expression of the cell cycle inhibitor p21, a p53 target, was only mildly increased (S6B and S6C Fig). Since p53 is activated in response to DNA damage, we performed immunofluorescence staining with an antibody against phosphorylated histone 2A (γH2AX), which recognizes DNA double strand breaks. Indeed, *ko*^*G*^ mice had significantly elevated levels of γH2AX positive cells in the epidermis. A mild increase was also seen in *ko*^*N*^ mice, but the loss of Nrf2 did not further aggravate the DNA damage that occurred in the absence of Gclc (Fig 6B).

Ultrastructural analysis of the epidermis from *ko*^*G*^ mice revealed that most of the apoptotic cells were located in the basal layer (Fig 6C). In addition, mitochondria, which depend on cytosolic GSH production for their integrity, were severely condensed, reflecting their damage (Fig 6D). This abnormality was particularly obvious in apoptotic keratinocytes (Fig 6D, middle panel), but even detectable in pre-apoptotic cells with features resembling ferroptosis, an iron-dependent form of non-apoptotic cell death [27] (Fig 6D, right panel). This likely results from oxidative stress as suggested by enhanced protein carbonylation, which reflects their oxidation state (Fig 6E).

**Fig 7:**
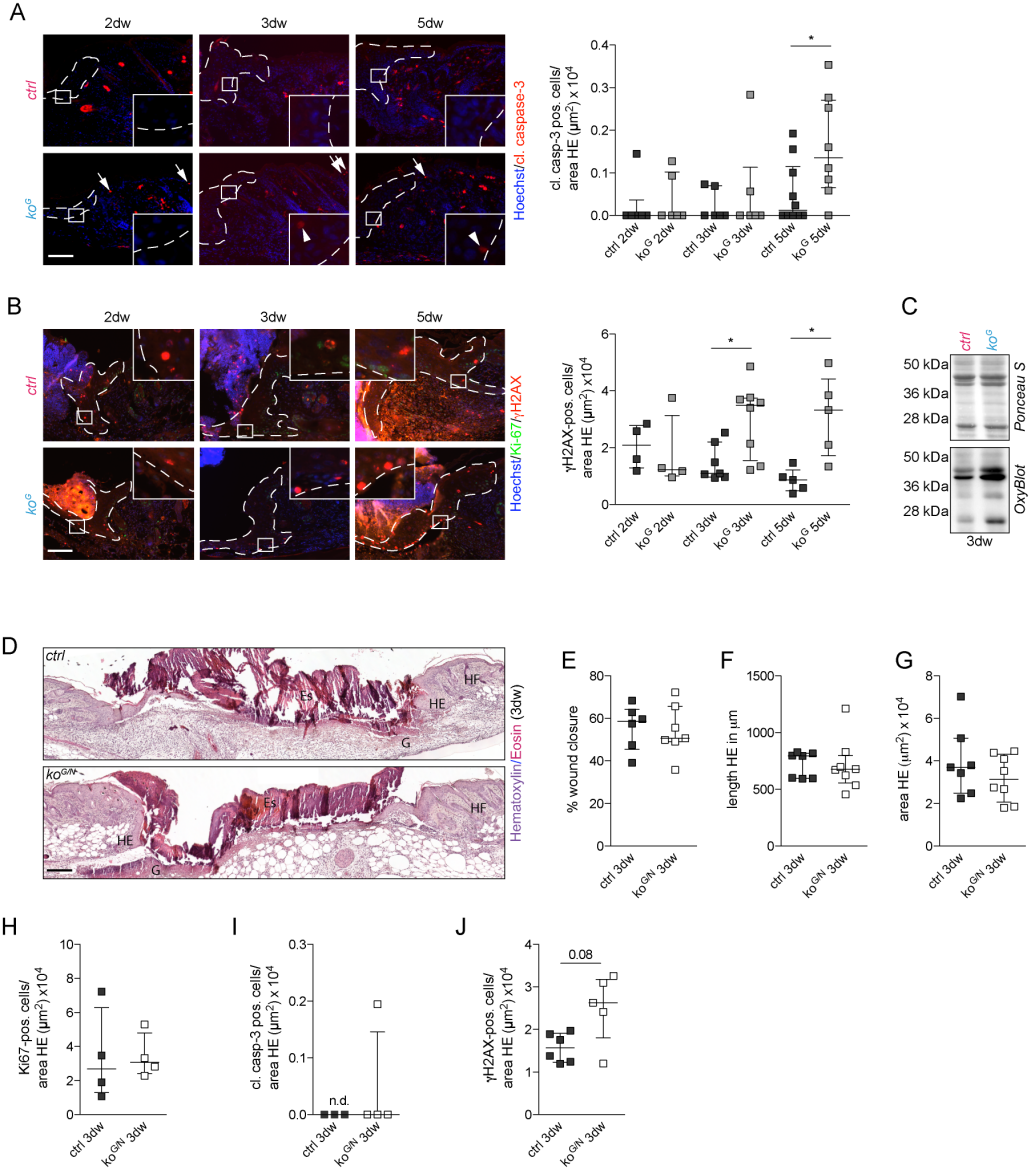
Cell damage and apoptosis in the hyperproliferative wound epithelium of Gclc-deficient mice. (A,B) Immunofluorescence-stained sections of 2-, 3- and 5-day wounds and quantification of (A) cleaved caspase-3 (arrowheads; arrows: positive cells in the adjacent epidermis), and (B) γH2AX positive cells per area HE. White dotted lines show the area of HE in which positive cells were counted. Each picture includes a magnification of the indicated square to show positive cells (if existing). Scale bar: 100 μm. For (A): 2dw N = 6; 3dw N = 6; 5dw N = 10/8. For (B): 2dw N = 4; 3dw N = 7/8; 5dw N = 5. (C) OxyBlot of lysates (pooled from 2 wounds of individual mice) from 3-day wounds. Equal loading was confirmed by Ponceau S staining of the membrane. (D) H&E staining on sections from 3-day wounds. Scale bar: 200 μm. D: dermis Es: eschar, G: granulation tissue, HF: hair follicle, HE: hyperproliferative epithelium. (E-G) Morphometric analysis of (E) percentage wound closure, (F) length HE and (G) area HE of 3-day wounds of *ko*^*G/N*^ and control mice. For (E): N = 6/7; for (F): N = 7/8; for (G): N = 7/8. (H-J) Quantification of (H) Ki-67 (N = 4), (I) cleaved caspase-3 (N = 3/4), and (J) γH2AX (N = 6/5) positive cells per area HE. Scatter plots show the median with interquartile range. *P ≤ 0.05. n.d.: not detectable.

Within the first 2–3 days after skin injury, the number of cleaved caspase-3 positive cells in the wound tongue was not significantly different between mice of both genotypes, but a strong increase was observed at day 5 post-injury in the wound epidermis of *ko*^*G*^ mice (Fig 7A). This finding provides a likely explanation for the reduced area of the wound epidermis that we detected at this time point (Fig 4E). Gclc-deficient wound keratinocytes also exhibited enhanced DNA double strand breaks (Fig 7B), concomitant with increased levels of carbonylated proteins in 3-day wounds (Fig 7C).

Combined loss of Nrf2 and Gclc had no further effect on the wound healing process, and the percentage of wound closure as well as length and area of the wound epithelium were not affected in the double knockout mice at day 3 after wounding compared to control mice (Fig 7D–7G). Similar to the situation in non-wounded skin, loss of Nrf2 did not affect proliferation and did not further increase the number of cleaved caspase-3 and γH2AX positive cells in the hyperproliferative wound epidermis (Fig 7H–7J).

### Apoptosis and DNA damage are direct consequences of GSH deficiency in keratinocytes

**Fig 8:**
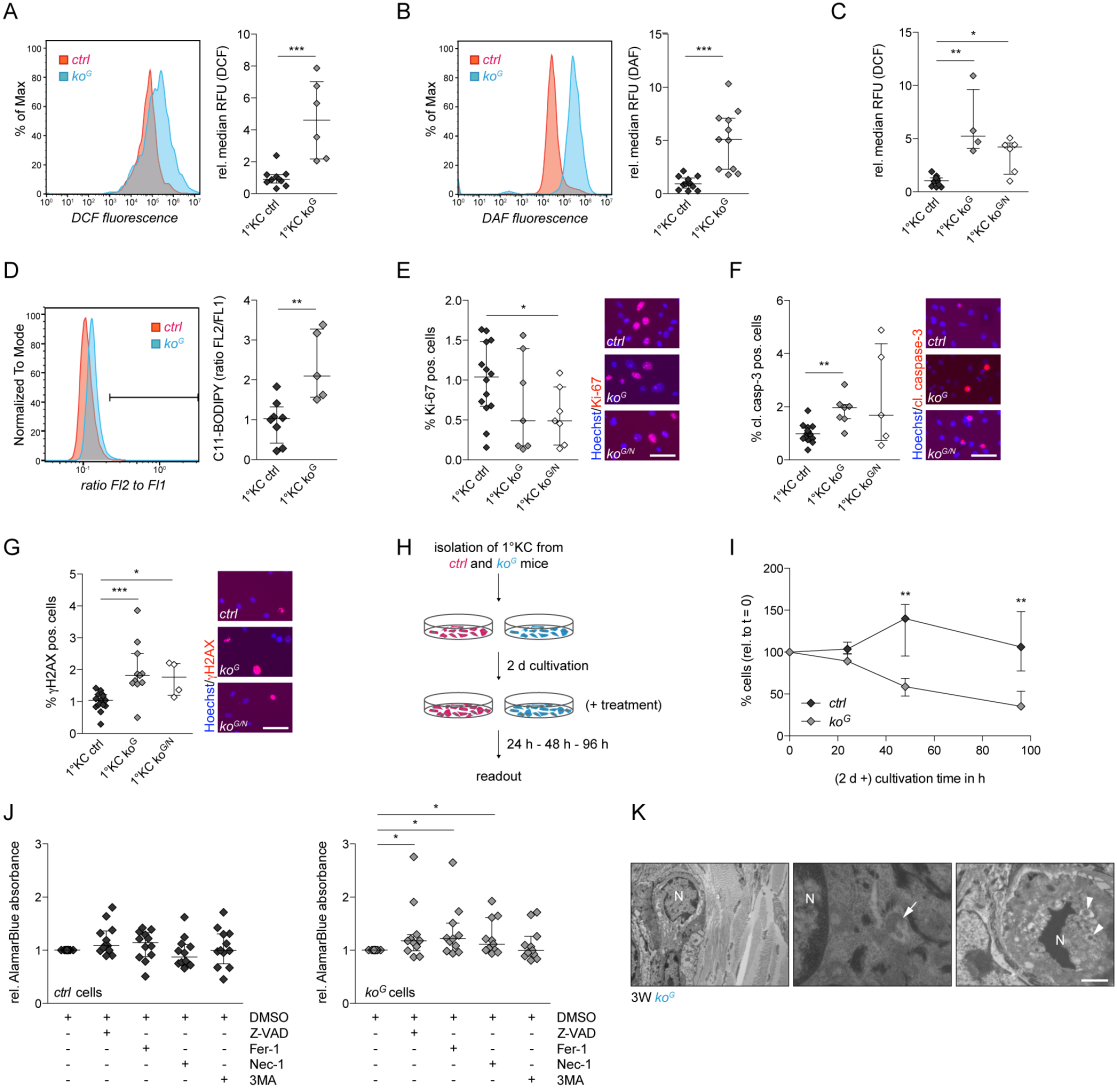
Gclc deficiency in keratinocytes causes cell damage through increased levels of ROS and RNS. (A,B) Representative histograms and quantification of relative median of relative fluorescence units (RFU) of (A) H_2_DCF-DA (N = 10/6) and (B) DAF-FM-DA (N = 13/11) assay of primary keratinocytes from *ctrl* and *ko*^*G*^ mice. (C) Quantification of RFU of H_2_DCF-DA assay of primary keratinocytes from *ctrl*, *ko*^*G*^ and *ko*^*G/N*^ mice. N = 11/4/6. (D) Representative histogram and quantification of a lipid peroxidation assay with the lipid peroxidation sensor C11-BODIPY^581/591^ (N = 8/5). (E-G) Immunofluorescence staining of primary keratinocytes and quantification of Ki-67 (E; N = 14/7/7), cleaved caspase-3 (F; N = 16/7/5)) and γH2AX (G; N = 15/11/4) positive cells. Scale bar: 50 μm. (H) Schematic representation of the experimental setup for (I,J). (I) Percentage of primary keratinocytes from individual mice after 24 h, 48 h and 96 h of cultivation in normal medium relative to number of cells at t = 0. N = 6. (J) AlamarBlue cell viability assay after treatment of primary keratinocytes from control or *ko*^*G*^ mice with DMSO (vehicle), Z-VAD, Fer-1, Nec-1 or 3MA in DMSO for 24 h. N = 12/11, analyzed in three independent experiments. Scatter plots show the median with interquartile range. *P ≤ 0.05, **P ≤ 0.01, ***P ≤ 0.001. (K) Electron microscopy of the epidermis at 3W. Left and middle panel: Keratinocytes with signs of ferroptotic cell death, including dysmorphic mitochondria and small mitochondria with increased membrane density and/or membrane accumulation. Right panel: Keratinocyte with signs of necroptotic cell death, including nuclear condensation, vacuolization and presence of autophagosomes. N = nucleus; arrowheads: autophagosomes; arrow: mitochondria. Scale bars: 5 μm (left panel), 700 nm (middle panel), or 3 μm (right panel).

To determine if DNA damage and apoptosis are direct consequences of GSH deficiency in keratinocytes, we analyzed primary keratinocytes during the first days after plating. Levels of ROS and RNS were significantly increased in Gclc-deficient keratinocytes (Fig 8A and 8B), and additional loss of Nrf2 did not further enhance the intracellular ROS levels (Fig 8C). Oxidative stress in these cells was further reflected by enhanced lipid peroxidation, which was detected with the lipid peroxidation sensor C11-BODIPY^581/591^ (Fig 8D).

The lack of Gclc caused a mild, but non-significant reduction in keratinocyte proliferation. The reduction was statistically significant for keratinocytes from the double knockout mice (Fig 8E), further confirming that the keratinocyte hyperproliferation observed *in vivo* at 2M is not a cell autonomous effect, but rather a consequence of increased inflammation. The number of cleaved caspase-3 and γH2AX positive cells was significantly increased in cultures from *ko*^*G*^ mice compared to controls, but—like in the *in vivo* situation—there was no further increase in cells from double mutant mice (Fig 8F and 8G). Therefore, DNA damage and the subsequent initiation of apoptosis are cell autonomous effects, which are directly triggered by the loss of GSH synthesis in keratinocytes and not amplified by loss of Nrf2.

Since we found a rapid decline in the number of Gclc-deficient keratinocytes upon culturing (Fig 8H and 8I), we determined if these cells die exclusively from apoptosis, or if non-apoptotic forms of cell death are also involved as suggested by the ferroptosis features that we detected in the Gclc-deficient epidermis by ultrastructural analysis (Fig 6D, right panel). For this purpose, Gclc-deficient primary keratinocytes and cells from control mice were incubated for 24 h with the pan-caspase inhibitor Z-VAD-FML (Z-VAD), the necroptosis inhibitor necrostatin-1 (Nec-1), the ferroptosis inhibitor ferrostatin-1 (Fer-1) or the autophagy inhibitor 3-methyladenine (3MA) (Fig 8H and 8J). None of these inhibitors affected the viability of cells from control mice (Fig 8J, left panel). However, the progressive cell death of keratinocytes from Gclc-deficient mice was partially rescued by low concentrations of Z-VAD, confirming that the cells die at least in part via apoptosis. Moreover, there was an increase in cell viability in the presence of necrostatin-1 or ferrostatin-1 (Fig 8J, right panel), indicating a contribution of necroptosis and ferroptosis to the cell death of *ko*^*G*^ primary keratinocytes. A more detailed ultrastructural analysis of the epidermis at 3W confirmed the presence of cells with signs of ferroptosis and also revealed cells with necroptosis-like features. This included dysmorphic mitochondria, as well as small mitochondria with increased membrane density and/or membrane accumulation, characteristic for ferroptosis (Fig 8K left and middle panel; Fig 6D, right panel) and nuclear condensation, severe vacuolization and autophagosome formation, characteristic for necroptosis (Fig 8K, right panel). These findings demonstrate the *in vivo* relevance of the functional cell culture studies and further suggest that Gclc-deficiency initiates different cell death pathways.

### Cysteine and thioredoxin reductase compensate for the loss of Gclc

**Fig 9:**
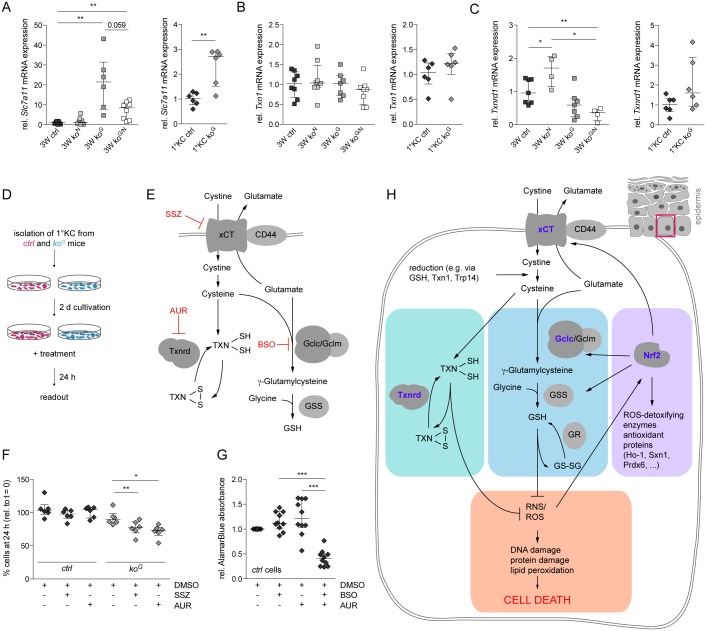
Slc7a11 and the thioredoxin/thioredoxin reductase system partially compensate for the loss of Gclc. (A-C) qRT-PCR of *Slc7a11* (A), *Txn1* (B) and *Txnrd1* (C) relative to *Rps29* using RNA form the epidermis at 3W (N = 7/8/6/8 for *Slc7a11*; N = 8/8/7/8 for *Txn1*, and N = 7/4/7/4 for *Txnrd1*) and from primary keratinocytes (N = 6). (D) Schematic representation of the experimental setup for (F,G). (E) Schematic representation of the mechanisms of action of the chemical inhibitors used in this study. SSZ inhibits the import of cystine by the xCT transporter, AUR inhibits the catalytic activity of Txnrd, and BSO inhibits the catalytic activity of Gcl. (F) Percentage of primary keratinocytes from individual mice treated with DMSO (vehicle), SSZ or AUR in DMSO for 24 h. N = 6. (G) AlamarBlue cell viability assay after treatment of primary keratinocytes from control mice with DMSO (vehicle), AUR, and/or BSO in DMSO for 24 h. N = 10, analyzed in three independent experiments. Scatter plots show the median with interquartile range. *P ≤ 0.05, **P ≤ 0.01, ***P ≤ 0.001. (H) Schematic representation of the GSH, Txn/Txnrd and Nrf2 antioxidant defense systems and their cross-talk in the epidermis. xCT (Slc7a11), which is stabilized by the CD44 protein, mediates import of cystine and concomitant export of glutamate. Cystine is reduced in the cytoplasm by GSH and most likely by Txn1 or thioredoxin-related protein 14 (Trp14). Oxidized Txn1 or Trp14 are reduced by Txnrd1. Cysteine is used for GSH biosynthesis or incorporated into proteins, including Txn1. Nrf2 is activated upon GSH deficiency and induces the expression of xCT, of enzymes involved in GSH production and recycling (Gclc, gclm, glutathione synthetase (GSS), glutathione reductase (GR)) and of various ROS-detoxifying and antioxidant proteins. Txn/Txnrd, GSH and Nrf2 activities result in a reduction of intracellular ROS and RNS, thereby preventing DNA and protein damage and ultimately cell death.

We next tested if other thiols can compensate at least in part for the loss of GSH in keratinocytes. An important candidate is free cysteine, which is mainly provided by the uptake of cystine through the cystine-glutamate exchange protein (Slc7a11; xCT) [28] and concomitant intracellular reduction to cysteine or by direct uptake through the Slc1a4 (Asc) transporter. Interestingly, a strong increase in *Slc7a11* expression was observed in the epidermis and in cultured keratinocytes of *ko*^*G*^ mice (Fig 9A), and a mild, but non-significant increase in *Slc1a4* expression was seen in the epidermis (S7A Fig). *Slc7a11* is a known Nrf2 target gene [29], and the increase in expression of this gene that occurred in the absence of Gclc was indeed partially rescued by loss of Nrf2. Nevertheless, expression levels of *Slc7a11* were still significantly elevated in *ko*^*G/N*^ mice compared to controls (Fig 9A). Expression levels of *Slc1a4*, which has not been described as an Nrf2 target, were not affected by the loss of Nrf2 only, but significantly reduced in the double knockout mice compared to *ko*^*G*^ mice (S7A Fig).

The intracellular reduction of cystine to cysteine requires GSH or potentially thioredoxin reductase (Txnrd) dependent systems [30–32], and the GSH and Txn/Txnrd systems were shown to be partially redundant in embryonic stem cells, cancer cells and also in mouse liver [23, 31, 33]. *Txn1* mRNA levels in the epidermis of young mice or in primary keratinocytes were not affected by the loss of Gclc (Fig 9B), but a mild increase was seen in the epidermis of mice at the age of 2M (S7B Fig). *Txnrd1* expression was mildly reduced in *ko*^*G*^ mice, and the reduction was statistically significant in *ko*^*G/N*^ mice (Fig 9C). However, the levels of this protein were similar in epidermal lysates from control and *ko*^*G*^ mice (S7C and S7D Fig). The difference in *Txnrd1* expression was no longer detectable at the age of 2M (S7E Fig). Txnrd2 expression was not affected by the loss of Gclc, but a mild increase in Txn2 protein levels was observed at the age of 3W (S7C and S7D Fig).

To determine if the Slc7a11/Txn/Txnrd system compensates for the loss of Gclc, we treated primary keratinocytes from Gclc-deficient and control mice with chemical inhibitors of Slc7a11 (sulfasalazine—SSZ) or Txnrd (auranofin—AUR) for 24 h (Fig 9D and 9E). While the viability of control cells was not or only mildly affected by these compounds, they caused a significant reduction in the number of viable Gclc-deficient keratinocytes (Fig 9F). The same tendency was seen upon staining for cleaved caspase-3 (S7F Fig). The detrimental effect of AUR on Gclc-deficient cells was verified in an alamarBlue survival assay (S7G Fig). To exclude a general susceptibility of Gclc knockout keratinocytes to chemical inhibitors, we analyzed inhibitor-treated keratinocytes from wild-type mice. While neither inhibition of Txnrd1 by AUR nor GSH depletion by BSO alone affected survival of these cells, a combination of both inhibitors severely affected their viability (Fig 9G), demonstrating that both systems have complementary protective functions in keratinocytes. These results provide insight into the roles of different antioxidant defense systems in keratinocytes and their orchestrated interplay *in vitro* and *in vivo* (Fig 9H) and led us to propose the following scenario: Loss of Gclc and subsequent glutathione deficiency in keratinocytes results in activation of the Nrf2 transcription factor as shown by the genetic approach in this study and by pharmacological GSH depletion [22]. However, in the absence of Gclc, Nrf2 can no longer activate GSH production, and various GSH-dependent Nrf2 targets are increased, but remain non-functional. The upregulation of Nrf2 target genes does not compensate for the loss of Gclc in *ko*^*G*^ mice as demonstrated by the lack of additional abnormalities in *ko*^*G/N*^ mice. By contrast, cystine, which is imported via the xCT transporter and intracellularly reduced to cysteine can partially compensate for the lack of GSH. Cysteine is incorporated into TXN, which can compensate for the lack of GSH in the reduction of cystine and as a ROS/RNS scavenger. Therefore, inhibition of the TXNRD pathway in Gclc-deficient keratinocytes causes massive cell death as shown in this study, whereas inhibition of either the GSH or the TXN pathway can be tolerated by keratinocytes *in vitro* and *in vivo*.

## Discussion

Previous studies revealed essential functions of GSH in development and organ homeostasis. Thus, mice with a global *Gclc* knockout die during embryonic development [4, 18], and hepatocyte-specific loss of Gclc causes liver failure within four weeks after birth [34]. Unexpectedly, we show here that most mice lacking Gclc in keratinocytes survive for several weeks or even months, most likely due to a remarkable redundancy in the antioxidant defense capacity of keratinocytes combined with a high regenerative capability of the epidermis.

### Loss of Gclc in keratinocytes causes mild macroscopic and histological abnormalities

The most obvious phenotype of the Gclc-deficient mice was the failure to gain weight after weaning. This is likely to result from impaired food uptake due to the severe hyperkeratosis in the forestomach. The deficit in weight gain may be further promoted by the progressive increase in TEWL. Malnutrition as the cause of cachexia and premature death in *ko*^*G*^ mice is further supported by their low blood glucose levels. By contrast, systemic effects are unlikely to contribute to the lack of weight gain and the reduced life expectancy since: (i) serum levels of Il-6 were below detection levels, (ii) expression of important cytokines, which could cause cachexia or other systemic abnormalities was not increased in the skin of the mutant mice, and (iii) Gclc expression was not affected in tissues where the K5 promoter is not active, such as liver and kidney.

The epidermis of Gclc-deficient mice showed hyperkeratosis and reduced adhesiveness of the corneocytes, providing a likely explanation for the mildly enhanced TEWL. This increase was followed by an elevation in the number of dermal mast cells and epidermal γδ T cells, suggesting that the accumulation of these immune cells is a consequence of the barrier defect. Consistent with this assumption, an increase in mast cells and epidermal γδ T cells is frequently observed in mice with impaired barrier function [35]. The mild inflammation may be further promoted by the early onset of apoptotic and also non-apoptotic cell death in the epidermis. The latter had previously been demonstrated to result in cutaneous inflammation [36]. Mast cells and epidermal γδ T cells produce a variety of growth factors and cytokines [37, 38], providing a possible explanation for the keratinocyte hyperproliferation that we observed in Gclc-deficient mice upon aging.

The cutaneous abnormalities in *ko*^*G*^ mice showed some similarities with the phenotype of mice lacking Gpx4 in keratinocytes, including cutaneous inflammation [39]. However, the inflammatory response was much milder, and the increase in dermal macrophages and neutrophils as well as the enhanced expression of various pro-inflammatory cytokines and of *Ptgs2/Cox2* observed in Gpx4-mutant mice was not seen in *ko*^*G*^ mice. Furthermore, there was no increase in the viable part of the epidermis in *ko*^G^ mice. The milder phenotype may be explained by the fact that Gpx4 can also use cysteine or other thiols as substrates [40]. Gpx4-deficient mice exhibit major hair follicle abnormalities and they develop transient baldness [39]. Hair loss was also seen in Gclc-deficient mice, but it was restricted to a few areas. In addition, abnormal hair follicles developed in mice lacking both Gclc and Nrf2, which were highly reminiscent to those observed in Gpx4-mutant mice. These findings suggest that loss of Nrf2 reduces the availability of other thiol substrates of Gpx4, which may then become rate limiting in the rapidly growing hair follicle keratinocytes.

### GSH protects keratinocytes from DNA damage and cell death

Ultrastructural and molecular analysis of the epidermis from Gclc-deficient mice revealed a strong increase in the number of apoptotic and pre-apoptotic cells. This is likely to result from the severe ROS- and RNS-induced DNA and mitochondrial damage. In addition, it is possible that certain proteins involved in the control of apoptosis are glutathionylated in wild-type mice and that the loss of such posttranslational modifications in *ko*^*G*^ mice affects their functions. This should be determined in the future using proteomics and follow-up functional approaches.

Our ultrastructural data combined with the functional *in vitro* studies strongly suggest that Gclc-deficient keratinocytes not only die from apoptosis, but that different forms of regulated necrosis are also involved, such as necroptosis and ferroptosis. The latter is consistent with an at least partial functional inhibition of Gpx4 in the absence of GSH, since Gpx4 was previously identified as a crucial regulator of ferroptotic cell death in tumor cells and kidney tubular cells [41, 42]. Gclc-deficient keratinocytes proliferated normally *in vivo*, suggesting remarkable compensatory mechanisms in this cell type that are even sufficient in response to skin injury. We did not even observe an increase in apoptosis in Gclc-deficient mice during the early phase of wound healing, possibly due to the upregulation of various growth and survival factors in healing skin wounds [20]. This is different to the severe wound healing defect in rats after BSO-mediated depletion of GSH in the wound tissue [15]. It may well be that cells of the dermis/granulation tissue are more dependent on GSH than keratinocytes and/or that the cell death induced by the lack of GSH in the epidermis is compensated for by the high regenerative capacity of the epidermis. An important role of GSH in granulation tissue formation is supported by the early onset of senescence in fibroblasts lacking the Gcl modifier subunit [43]. Alternatively, long-term GSH deficiency may allow upregulation of compensatory mechanisms that cannot come in place upon acute depletion.

### Activation of Nrf2 does not compensate for the loss of Gclc in keratinocytes

Several lines of evidence suggested a key role of Nrf2 in the compensation of GSH deficiency: (i) Nrf2 target genes were upregulated in Gclc-deficient keratinocytes. This is in line with the induction of Nrf2 activity by GSH deficiency and the identification of Nrf2 as a functional adaptive response to GSH depletion in neuroblastoma cells and fibroblasts [22, 44]; (ii) Nrf2 induces the expression of Slc7a11 [29], the cystine transporter, which had rescued the death of Gclc-deficient embryonic stem cells upon forced overexpression [31]; (iii) hyperkeratosis was also observed in the epidermis and the hair follicle infundibulum of mice expressing a caNrf2 mutant in keratinocytes [25]; (iv) caNrf2-transgenic as well as *ko*^*G*^ mice had a defect in the epidermal barrier, most likely as a result of abnormal expression of Sprr2d, a major component of the cornified envelope [25], and subsequent alterations in the expression of lipid biosynthesis enzymes such as Elovl3 [25]; and (v) activation of Nrf2 rescued the barrier defect in loricrin knockout mice and thus prevented postnatal lethality [45]. However, our genetic data revealed that Nrf2 is not acting as a back-up system to compensate for the lack of Gclc in keratinocytes. The most likely explanation is that any cytoprotective effect of Nrf2 is mediated via its strong induction of enzymes involved in GSH synthesis and recycling. In the absence of Gclc, this protective effect is no longer in place, and the upregulation of Nrf2 in Gclc-deficient cells can therefore not result in a functional rescue. However, activation of Nrf2 is likely to have a protective effect when GSH depletion is not the consequence of a loss of its biosynthesis enzymes. In this case, Nrf2 will promote their expression, resulting in restoration of normal GSH levels.

### Thioredoxin and GSH act independently to protect keratinocytes from oxidative damage

While activation of Nrf2 is obviously not responsible for the relatively mild phenotype of *ko*^*G*^ mice, the upregulation of Slc7a11 that occurred at least in part in an Nrf2-independent manner, was more important, and pharmacological inhibition of this transporter induced death of Gclc-deficient keratinocytes. An even more striking result was obtained with a Txnrd inhibitor, which strongly reduced the viability of cultured Gclc-deficient keratinocytes and also promoted death of wild-type keratinocytes when applied together with a Gclc inhibitor. Therefore, the Txn/Txnrd system indeed compensates at least in part for the loss of GSH, most likely through the capacity to reduce the imported cystine. These results are consistent with findings obtained with embryonic stem cells or cancer cells, which demonstrated that loss of GSH can be compensated for by the Txn/Txnrd system and *vice versa* [23, 31, 46]. Importantly, we show here that this compensatory activity is relevant in a normal tissue *in vivo* without the need of forced overexpression of one component. Our results also highlight the remarkably diverse antioxidant capacity of keratinocytes, which allows them to survive in response to various insults and to maintain the epidermal barrier function, which guarantees whole body homeostasis even under stress conditions. The compensatory function of thioredoxin also provides an explanation for the resistance of epidermal keratinocytes to various anti-cancer drugs, of which many affect GSH metabolism [47]. This allows maintenance of skin integrity in the treated cancer patients. Finally, our results provide important insight into the interplay between different cellular antioxidant defense systems.

## Materials and Methods

### Animals

Mice expressing Cre recombinase under control of the keratin 5 promoter (K5Cre mice) [19] were mated with mice harboring floxed alleles of the *Gclc* gene [34] and/or floxed alleles of the *Nrf2* gene [48]. All mice were in C57Bl/6 genetic background. They were housed under optimal hygiene conditions and maintained according to Swiss animal protection guidelines. All experiments with animals were approved by the “Kantonales Veterinäramt Zürich” (CH 32/2011 and ZH 247/2013). The work was performed in strict accordance with the Schweizerisches Tierschutzgesetz (Animal Welfare Act) and the subordinate Tierschutzordnung (Animal Welfare Ordinance) and Tierversuchsverordnung (Animal Experimentation Ordinance).

### Wounding and preparation of wound tissue

Mice between P19 and P24 were anaesthetized by inhalation of 2% isoflurane. One full-thickness excisional wound, 4 mm in diameter, was made on each side of the dorsal midline by excising skin and *panniculus carnosus*. Mice were sacrificed at different time points after injury. For histological analysis, the complete wounds were excised and either fixed overnight in 95% ethanol/1% acetic acid or in 4% paraformaldehyde / phosphate buffered saline (PBS) followed by paraffin embedding, or directly frozen in tissue freezing medium (Leica Microsystems, Heerbrugg, Switzerland). Sections (7 μm) from the middle of the wounds were stained with H&E or used for immunofluorescence analysis. Morphometric analysis of different parameters of the wound healing process was performed using H&E- or immunofluorescence-stained sections as shown schematically in Fig 4A.

### Measurement of blood glucose levels

Blood glucose levels were determined using a commercial blood glucose meter.

### Separation of dermis from epidermis of mouse back skin

Separation of epidermis from dermis was achieved either by heat shock treatment (30 s at 55–60°C followed by 1 min at 4°C, both in PBS), by incubation for 50–60 min at 37°C in 0.143% dispase/DMEM or by incubation in 0.8% trypsin/DMEM for 15–30 min at 37°C. For dispase and trypsin treatment the subcutaneous fat was gently scraped off with a scalpel prior to incubation. Isolated epidermis was either directly snap frozen and stored at -80°C, or homogenized and further processed.

### Analysis of TEWL

TEWL was determined using a Tewameter (Courage and Khazaka Electronic GmbH, Cologne, Germany). The probe was placed on the dorsal back skin of shaved mice and measurements were performed for 30–50 consecutive counts on two different spots.

### RNA isolation and qRT-PCR

Total RNA from tissue was isolated with Trizol followed by purification with the RNeasy Mini Kit, including on-column DNase treatment (Qiagen, Hilden, Germany). Total RNA from cells was extracted directly with the RNeasy Mini Kit. cDNA was synthesized using the iScript kit (Bio-Rad Laboratories, Hercules, CA). Relative gene expression was determined using the Roche LightCycler 480 SYBR Green system (Roche, Rotkreuz, Switzerland). Primers used for qRT-PCR are shown in S1 Table.

### Cell culture

Keratinocytes were isolated from single mice as described previously [35] and cultured in a 7:5 mixture of keratinocyte serum-free medium (Invitrogen/Life Technologies, Carlsbad, CA) supplemented with 10 ng/ml EGF, 10^−10^ M cholera toxin and 100 U/ml penicillin/100 μg/ml streptomycin (Sigma) and of keratinocyte medium [49]. Plates were coated with collagen IV prior to seeding of the cells. For treatment with chemical inhibitors, primary keratinocytes were isolated from individual mice, cultured for 2 days, and incubated with the solvent DMSO (equal amount as in inhibitor samples), 15 mM BSO (Sigma), 250 μM SSZ (Sigma), 75 nM AUR (Sigma), 10 μM Z-VAD (Enzo Life Sciences, Lausen, Switzerland), 1 μM Fer-1 (Xcessbio, San Diego, CA), 20 μM Nec-1 (Enzo), or 10 μM 3MA (Sigma) for 24 h. Viable cells were either counted or analyzed using the alamarBlue cell viability reagent as described by the manufacturer (Invitrogen/Life Technologies).

### Preparation of protein lysates and western blotting

Frozen tissue was homogenized in T-PER tissue protein extraction reagent (Pierce, Rockford, IL) containing Complete Protease Inhibitor Cocktail (Roche). Lysates were cleared by sonication and centrifugation (13,000 rpm, 30 min, 4°C), snap frozen, and stored at -80°C. The protein concentration was determined using the BCA Protein assay (Pierce). Proteins were separated by SDS-PAGE and transferred onto nitrocellulose membranes. Antibodies used for Western blotting are listed in S2 Table.

### OxyBlot analysis

Oxidized proteins were detected using the OxyBlot assay kit (Chemicon, Temecula, CA) according to the manufacturer’s instructions. The method is based on the detection of protein carbonyl groups derivatized with 2,4-dinitrophenylhydrazine to convert carbonyl groups to dinitrophenylhydrazone derivatives. Derivatized protein samples were subjected to SDS-PAGE and subsequent Western blot analysis using an antibody against dinitrophenylhydrazone.

### Immunofluorescence and immunohistochemistry

After deparaffinization and rehydration or fixation with cold methanol (in case of frozen sections), unspecific binding sites were blocked with PBS containing 3–12% BSA and 0.025% NP-40 for 1 h at room temperature, and then incubated overnight at 4°C with the primary antibodies (see S3 Table) diluted in the same buffer. If needed, antigen retrieval was performed prior to the blocking procedure by cooking in citrate buffer (1 h at 95°C). For immunohistochemistry, endogenous peroxidase activity was quenched with 3% H_2_O_2_ for 10 min prior to blocking. After three washes with PBST (1 x PBS/0.1% Tween 20), slides were incubated at room temperature for 1 h with secondary antibodies and Hoechst (1 μg/ml) as counter-stain, washed with PBST again and mounted with Mowiol (Hoechst, Frankfurt, Germany). Sections were photographed using a Zeiss Imager.A1 microscope equipped with an Axiocam MRm camera and EC Plan-Neofluar objectives (10x/0.3, 20x/0.5). For data acquisition the Axiovision 4.6 software was used (all from Carl Zeiss, GmbH, Oberkochen, Germany). Antibodies used for immunostaining are listed in S3 Table.

### Staining of mast cells with toluidine blue

After deparaffinization and rehydration, skin sections were stained with 0.5% toluidine blue, 0.5 N HCl, pH 2.3 for 30 min. After washing with distilled water, sections were dehydrated and mounted with Eukitt mounting medium (Sigma). Stained mast cells appear violet or purple.

### Flow cytometry

Dermis and epidermis were separated as described above, further processed into single cell suspensions and stained as described previously [50]. Dyes and antibodies used for flow cytometry are listed in S4 Table. Fluorescence was directly measured by flow cytometry using the BD LSRFortessa (BD Biosciences, San Jose, CA).

### Electron microscopy

Mice were lethally anesthetized with pentobarbital (700 mg/kg) and perfused with 4% PFA in PBS. Skin samples were kept overnight in fixation solution, rinsed, and stored in PBS. After washing in 0.1 M cacodylate buffer pH 7.2 at 4°C, the specimens were treated with 2% OsO_4_ for 2 h. After washing, they were stained in 1% uranyl acetate, dehydrated and embedded in araldite resin. Ultra-thin sections (30–60 nm) were processed with a diamond knife and placed on copper grids. Transmission electron microscopy was performed using a 902A electron microscope (Zeiss, Oberkochen, Germany).

### Determination of GSH concentration

Cells or heat shock isolated epidermis were lysed in 0.6% sulfosalicylic acid/0.1% Triton-X100 in phosphate buffer containing 5 mM EDTA. After centrifugation, total GSH/GSSG levels in the supernatant were determined using the enzymatic recycling method [51].

### Determination of ROS, NO and lipid peroxidation

Levels of intracellular ROS and NO and extent of lipid peroxidation in primary murine keratinocytes were determined using H_2_DCF-DA (Invitrogen/Life Technologies), DAF-FM-DA (Sigma), or C11-BODIPY^581/591^ assays (Invitrogen/Life Technologies), respectively. H_2_DCF-DA allows detection of intracellular H_2_O_2_, but it also detects oxygen radicals [52]. DAF-FM-DA is a probe to detect NO, but it only works under aerobic conditions and it is likely to react with an oxidative product of NO, rather than with NO itself [53]. Cells were incubated for 30 min with 50 μM H_2_DCF-DA or 5 μM DAF-FM-DA, or for 2 h with 2 μM C11-BODIPY^581/591^ in cell culture medium at 37°C prior to detachment by trypsin. Fluorescence was directly measured by flow cytometry using the BD Accuri C6 (BD Biosciences, San Jose, CA).

### Statistical analysis

Statistical analysis was performed using the Prism4 software (GraphPad Software Inc, San Diego, CA). Significance was calculated with the Mann–Whitney *U* test for Non-Gaussian distribution or the Wilcoxon signed rank test. Log-rank test was used for the survival data.

## Supporting Information

S1 Fig Expression of GSH-dependent enzymes in the epidermis of *ko*^*G*^ mice, mild alterations in skin morphology in young *ko*^*G*^ mice and patchy hair loss of *ko*^*G*^ mice older than 3W. (A) Representative Western blot using lysates from epidermis of mice at 3W for peroxiredoxin 6 (Prdx6), glutathione peroxidase 4 (Gpx4), glutaredoxin 2 (Glrx2) and β-actin (loading control). (B) Quantification of protein expression levels. N = 4 for Prdx6 and Glrx2; N = 6 for Gpx4. (C) Pictures of *ko*^*G*^ and *ctrl* mice showing patchy hair loss at 2M (left panel, white square) and at 1.5M (right panel) in the knockout mice. (D) Picture of shaved *ko*^*G*^ and *ctrl* mice showing reduced flexibility of the skin in *ko*^*G*^ mice at 2M. (E) Microscopic pictures of isolated hairs from ctrl and *ko*^*G*^ mice at the age of 3W and 2M. Note the thinning and malformation of hairs from the mutant mice. Scale bar: 20 μm. (F) Density of hair follicles in the skin of *ko*^*G*^ and *ctrl* mice at the age of 3W and 2M (3W N = 11/10; 2M N = 6/7) (G) H&E staining of hair follicles in the skin of *ko*^*G*^ and *ctrl* mice at the age of 3W and 2M. Rectangles in the left panels show area selected for higher magnification in the right panels. Note the hyperkeratosis in the infundibula of *ko*^*G*^ mice (arrows), which results in narrowing of the hair canal and impaired anchorage of the hairs. Scale bar: 20 μm. Scatter plots show the median with interquartile range. *P ≤ 0.05.
(TIF)

S2 Fig Reduced serum glucose levels, but lack of obvious systemic abnormalities in *ko*^*G*^ mice. (A) Blood glucose levels of *ko*^*G*^ and control mice at the age of 3W and 2M (3W N = 17/7; 2M N = 9). (B) Macroscopic pictures of the incisors from *ko*^*G*^ and control mice at the age of 2M. Scale bar: 0.25 cm. (C) qRT-PCR of *Gclc* relative to *Rps29* using RNA from kidney and liver of *ko*^*G*^ and ctrl mice at the age of 2M (N = 6). Scatter plots show the median with interquartile range. *P ≤ 0.05 and **P ≤ 0.01.
(TIF)

S3 Fig Lack of skin abnormalities in young *ko*^*G*^ mice and normal processing of filaggrin. (A) H&E staining of longitudinal skin sections from mice at embryonic day 20 (E20), postnatal day 2.5 (P2.5), P7 and P18. Scale bar: 200 μm. (B) Western blot analysis for filaggrin using epidermal lysates of *ctrl* and *ko*^*G*^ mice at the age of 3W. Equal loading was confirmed by Ponceau S staining of the membrane.
(TIF)

S4 Fig Flow cytometric analysis of epidermal and dermal immune cells of control and *ko*^*G*^ mice. (A) Representative flow cytometry density plots to demonstrate gating of epidermal samples from *ctrl* and *ko*^*G*^ mice at 3W and 2M. Subsequent gating is shown from left to right. (B) Flow cytometry analysis of dermal cells from mice at 3W or 2M using different immune cell markers. 3W N = 8/6; 2M N = 5. (C) Representative flow cytometry density plots to demonstrate gating of dermal samples from *ctrl* and *ko*^*G*^ mice at 3W and 2M. Subsequent gating is shown from left to right—upper panel; F4/80^+^ and Ly6G^+^ cells were both gated from CD45^+^ cells.
(TIF)

S5 Fig Upregulation of Nrf2 target genes in *ko*^*G*^ epidermis at 2M and 3W and phenotype of *ko*^*G/N*^ mice at the age of 2M. (A) qRT-PCR of *Nqo1*, *Srxn1*, *Slpi* and *Sprr2d* relative to *Rps29* using RNA from the epidermis at 2M. *Nqo1*, *Slpi* and *Sprr2d*: N = 5. *Srxn1*: N = 5/4. (B) qRT-PCR of *Gsta3*, *Cbr1*, *Cbr3*, *and Aldh3a* relative to *Rps29* using RNA from the epidermis at 3W. *Gsta3*: N = 4. *Cbr1*, *Cbr3*, and *Aldh3a*: N = 5. (C) qRT-PCR of *Elovl1*, *Elovl3*, *Elovl4*, *Elovl5*, *Elovl6*, and *Elovl7* relative to *Rps29* using RNA from the epidermis at 3W and 2M. 3W N = 6; 2M N = 5. (D) qRT-PCR of catalase (*Cat*) and superoxide dismutase 1 (*Sod1*) relative to *Rps29* using RNA from the epidermis at 3W and 2M (left panel) and from primary keratinocytes (middle panel). 3W and 2M N = 5. 1°KC N = 6. qRT-PCR of L-gulonolactonoxidase (*Gulo*) relative to *Rps29* using RNA from the epidermis at 3W and from epidermis, total skin and liver at 2M (right panel). N = 3. (E) H&E staining of skin sections from mice at 2M demonstrating severe hyperkeratosis in *ko*^*G/N*^ mice. Scale bar: 40 μm. (F) Body weight of mice at 2M (N = 7/6). (G) Transepidermal water loss (TEWL) of mice at 2M (N = 7/6). Scatter plots show the median with interquartile range. *P ≤ 0.05 and **P ≤ 0.01.
(TIF)

S6 Fig Early onset of apoptosis in the epidermis of *ko*^*G*^ mice and p53 expression in the epidermis of *ko*^*G*^ mice at 3W and 2M. (A) Quantification of cleaved caspase-3 positive cells per length epidermis in immunofluorescence stained skin sections from control and *ko*^*G*^ mice at P2.5 and P7. P2.5: N = 4; P7: N = 5. (B) Immunofluorescence staining of skin sections at 3W (N = 8/9) and 2M (N = 5) for p53 and quantification of cells with p53-positive nuclei per length epidermis. Scale bar: 10 μm. (C) qRT-PCR analysis of *p21* relative to *Rps29* using RNA from the epidermis at 3W. N = 5. Scatter plots show the median with interquartile range. *P ≤ 0.05, **P ≤ 0.01, and ***P ≤ 0.001.
(TIF)

S7 Fig Expression of Txnrd1, Txnrd2 and Txn2 in the epidermis of *ko*^*G*^ mice and compensatory function of Xct and Txnrd in keratinocytes of *ko*^*G*^ mice. (A) qRT-PCR of *Slc1a4* relative to *Rps29* using RNA form the epidermis at 3W. N = 8/8/7/8. (B) qRT-PCR of *Txn1* relative to *Rps29* using RNA form the epidermis at 2M. N = 6/5/6/6. (C) Representative Western blot of lysates from mouse epidermis at 3W for Txnrd1, Txnrd2, Txn2 and β-actin (loading control). (D) Quantification of protein expression levels. N = 4 for Txnrd1 and Txnrd2; N = 6 for Txn2. (E) qRT-PCR of *Txnrd1* relative to *Rps29* using RNA form the epidermis at 2M. N = 6/5/6/6. (F) Quantification of percentage of cleaved caspase-3 positive cells in primary keratinocyte cultures from individual mice after 24 h incubation with DMSO (vehicle), SSZ or AUR (both in DMSO). N = 4. (G) AlamarBlue cell viability assay after treatment of primary keratinocytes with DMSO (vehicle) or AUR in DMSO for 24 h. N = 5, analyzed in two independent experiments. Scatter plots show the median with interquartile range. *P ≤ 0.05.
(TIF)

S1 Table Primers used for qRT-PCR. The sequences of all forward and reverse primers used for qRT-PCR are shown.
(PDF)

S2 Table List of antibodies used for western blotting. The antibodies used for western blotting are shown in the Table, including the antigen, the host in which the antibody was generated, the catalogue number, and the source.
(PDF)

S3 Table List of antibodies used for immunostaining. The antibodies used for immunostaining are shown in the Table, including the antigen, the host in which the antibody was generated, the catalogue number, and the source.
(PDF)

S4 Table List of dyes and antibodies used for flow cytometry. The dyes and antibodies used for flow cytometry are shown in the Table, including the antigen/dye, the fluorophore coupled to the dye/antibody, the catalogue number, and the source.
(PDF)
