# Supplementary figures and images for: A Glutathione-Nrf2-Thioredoxin Cross-Talk Ensures Keratinocyte Survival and Efficient Wound Repair
Source: PLoS Genet. 2016 Jan 25;12(1):e1005800. doi: 10.1371/journal.pgen.1005800 (PMC4726503; doi:10.1371/journal.pgen.1005800)

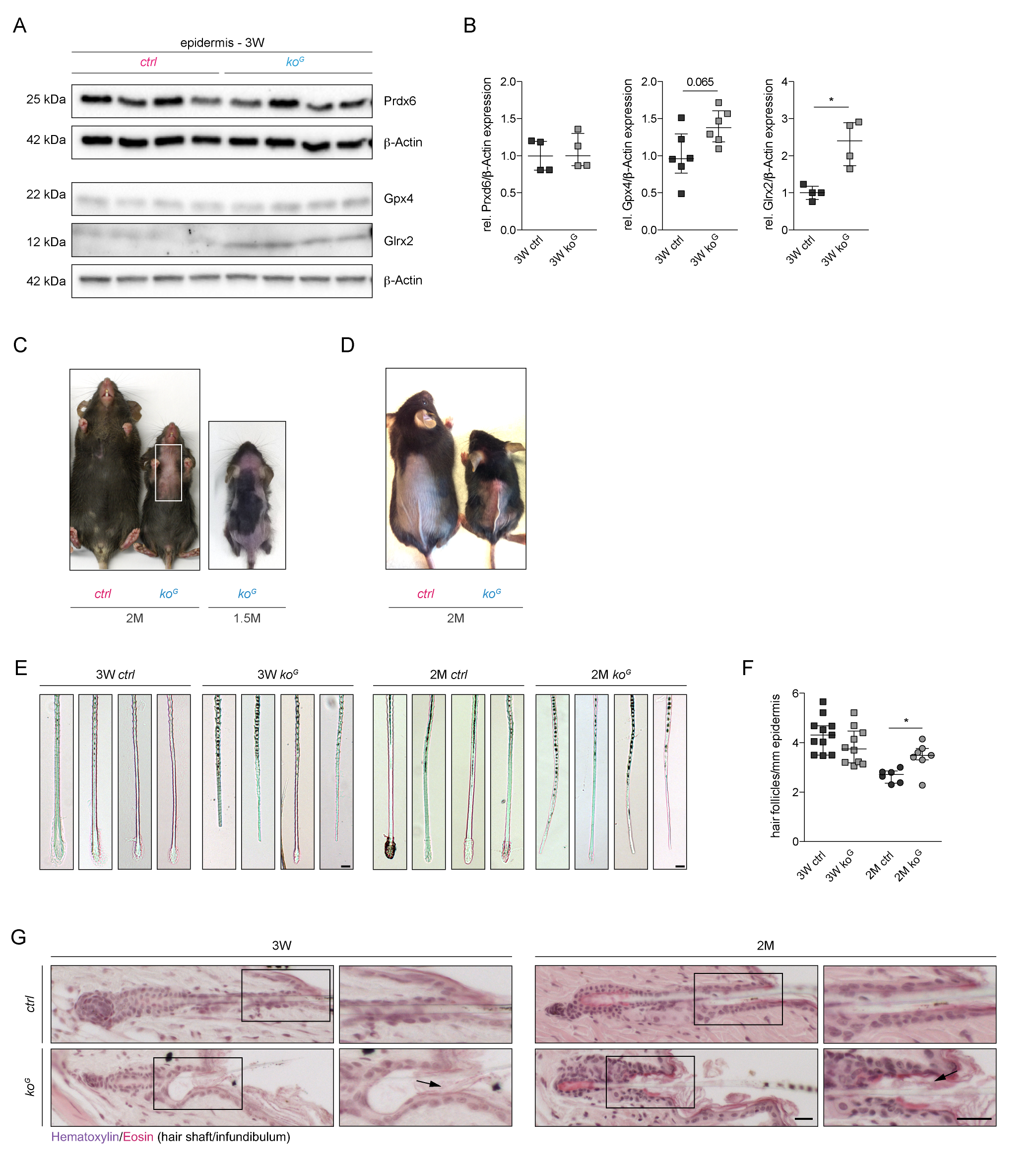

Supplement: S1 Fig — (A) Representative Western blot using lysates from epidermis of mice at 3W for peroxiredoxin 6 (Prdx6), glutathione peroxidase 4 (Gpx4), glutaredoxin 2 (Glrx2) and β-actin (loading control). (B) Quantification of protein expression levels. N = 4 for Prdx6 and Glrx2; N = 6 for Gpx4. (C) Pictures of koG and ctrl mice showing patchy hair loss at 2M (left panel, white square) and at 1.5M (right panel) in the knockout mice. (D) Picture of shaved koG and ctrl mice showing reduced flexibility of the skin in koG mice at 2M. (E) Microscopic pictures of isolated hairs from ctrl and koG mice at the age of 3W and 2M. Note the thinning and malformation of hairs from the mutant mice. Scale bar: 20 μm. (F) Density of hair follicles in the skin of koG and ctrl mice at the age of 3W and 2M (3W N = 11/10; 2M N = 6/7) (G) H&E staining of hair follicles in the skin of koG and ctrl mice at the age of 3W and 2M. Rectangles in the left panels show area selected for higher magnification in the right panels. Note the hyperkeratosis in the infundibula of koG mice (arrows), which results in narrowing of the hair canal and impaired anchorage of the hairs. Scale bar: 20 μm. Scatter plots show the median with interquartile range. *P ≤ 0.05. (TIF) [file pgen.1005800.s001.tif]

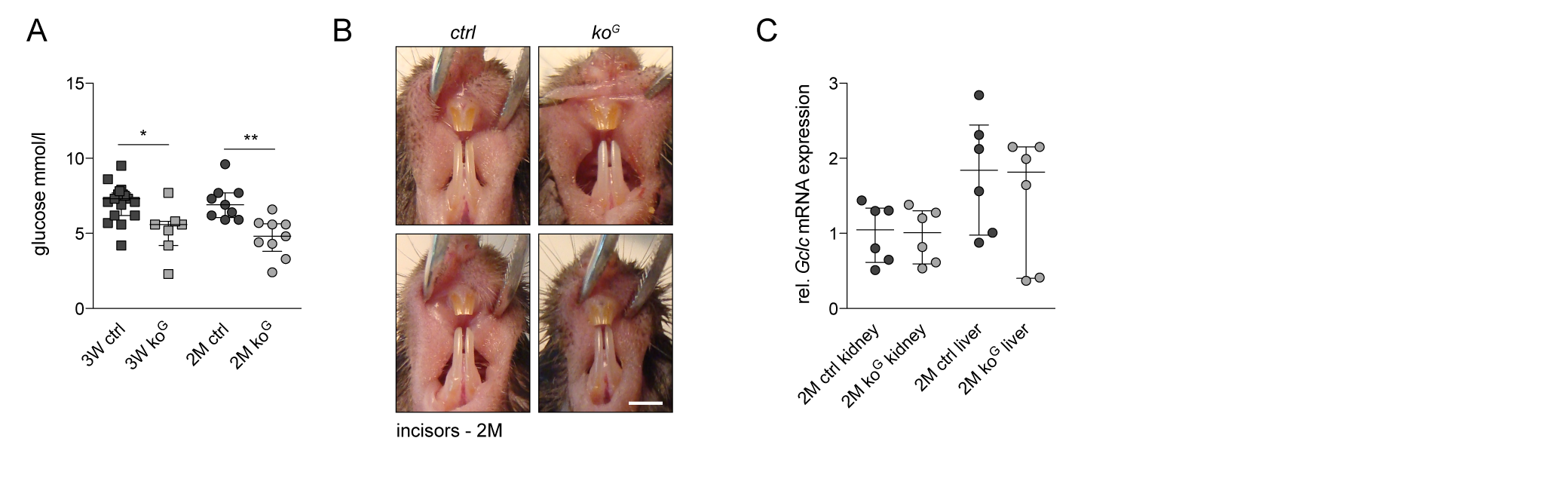

Supplement: S2 Fig — (A) Blood glucose levels of koG and control mice at the age of 3W and 2M (3W N = 17/7; 2M N = 9). (B) Macroscopic pictures of the incisors from koG and control mice at the age of 2M. Scale bar: 0.25 cm. (C) qRT-PCR of Gclc relative to Rps29 using RNA from kidney and liver of koG and ctrl mice at the age of 2M (N = 6). Scatter plots show the median with interquartile range. *P ≤ 0.05 and **P ≤ 0.01. (TIF) [file pgen.1005800.s002.tif]

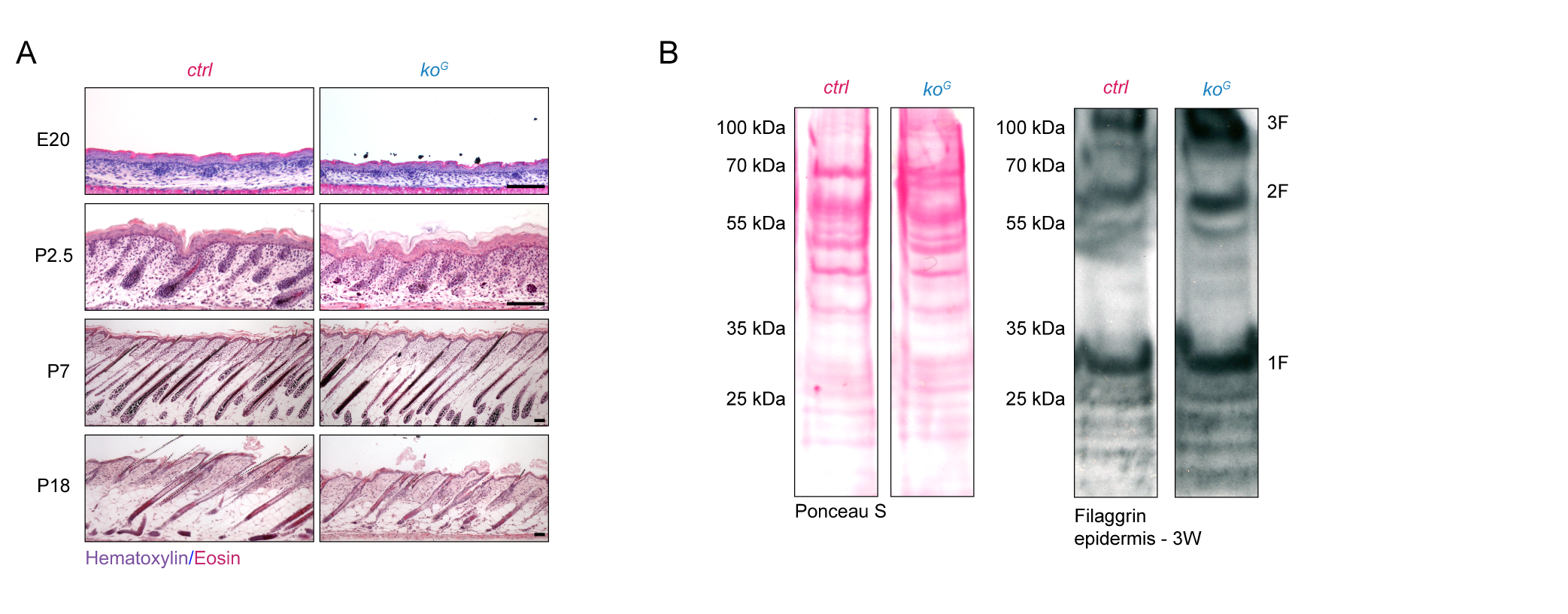

Supplement: S3 Fig — (A) H&E staining of longitudinal skin sections from mice at embryonic day 20 (E20), postnatal day 2.5 (P2.5), P7 and P18. Scale bar: 200 μm. (B) Western blot analysis for filaggrin using epidermal lysates of ctrl and koG mice at the age of 3W. Equal loading was confirmed by Ponceau S staining of the membrane. (TIF) [file pgen.1005800.s003.tif]

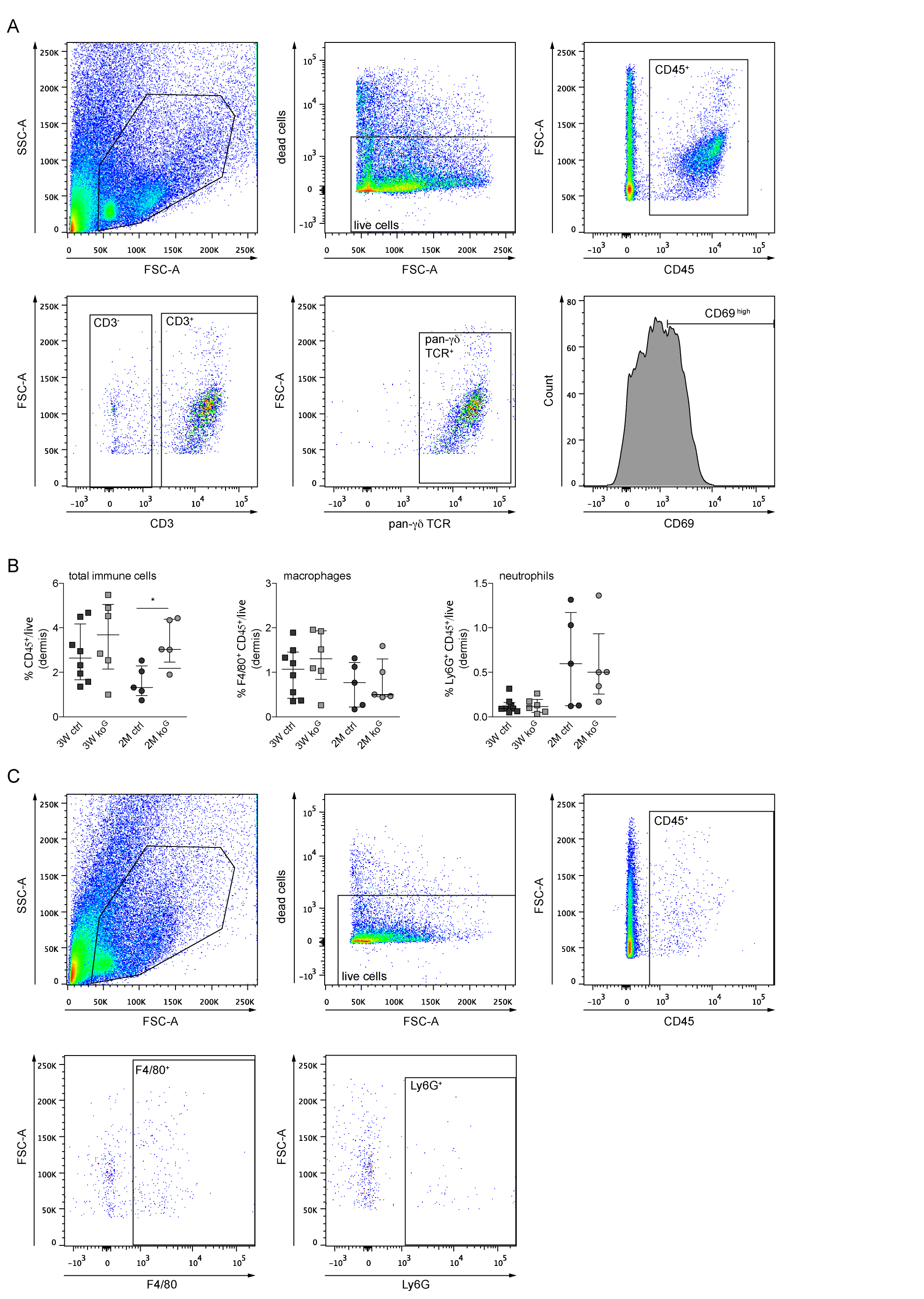

Supplement: S4 Fig — (A) Representative flow cytometry density plots to demonstrate gating of epidermal samples from ctrl and koG mice at 3W and 2M. Subsequent gating is shown from left to right. (B) Flow cytometry analysis of dermal cells from mice at 3W or 2M using different immune cell markers. 3W N = 8/6; 2M N = 5. (C) Representative flow cytometry density plots to demonstrate gating of dermal samples from ctrl and koG mice at 3W and 2M. Subsequent gating is shown from left to right—upper panel; F4/80+ and Ly6G+ cells were both gated from CD45+ cells. (TIF) [file pgen.1005800.s004.tif]

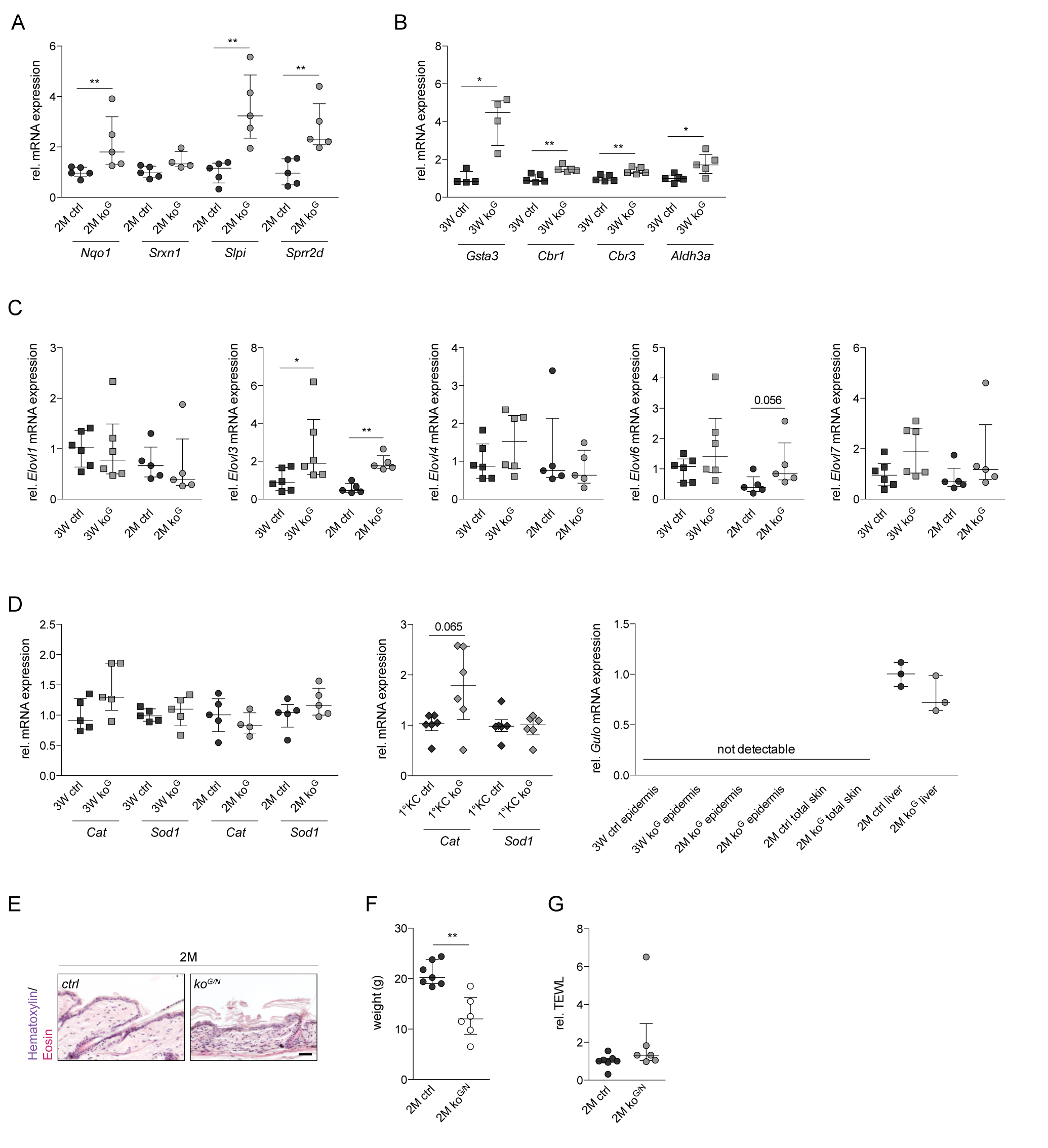

Supplement: S5 Fig — (A) qRT-PCR of Nqo1, Srxn1, Slpi and Sprr2d relative to Rps29 using RNA from the epidermis at 2M. Nqo1, Slpi and Sprr2d: N = 5. Srxn1: N = 5/4. (B) qRT-PCR of Gsta3, Cbr1, Cbr3, and Aldh3a relative to Rps29 using RNA from the epidermis at 3W. Gsta3: N = 4. Cbr1, Cbr3, and Aldh3a: N = 5. (C) qRT-PCR of Elovl1, Elovl3, Elovl4, Elovl5, Elovl6, and Elovl7 relative to Rps29 using RNA from the epidermis at 3W and 2M. 3W N = 6; 2M N = 5. (D) qRT-PCR of catalase (Cat) and superoxide dismutase 1 (Sod1) relative to Rps29 using RNA from the epidermis at 3W and 2M (left panel) and from primary keratinocytes (middle panel). 3W and 2M N = 5. 1°KC N = 6. qRT-PCR of L-gulonolactonoxidase (Gulo) relative to Rps29 using RNA from the epidermis at 3W and from epidermis, total skin and liver at 2M (right panel). N = 3. (E) H&E staining of skin sections from mice at 2M demonstrating severe hyperkeratosis in koG/N mice. Scale bar: 40 μm. (F) Body weight of mice at 2M (N = 7/6). (G) Transepidermal water loss (TEWL) of mice at 2M (N = 7/6). Scatter plots show the median with interquartile range. *P ≤ 0.05 and **P ≤ 0.01. (TIF) [file pgen.1005800.s005.tif]

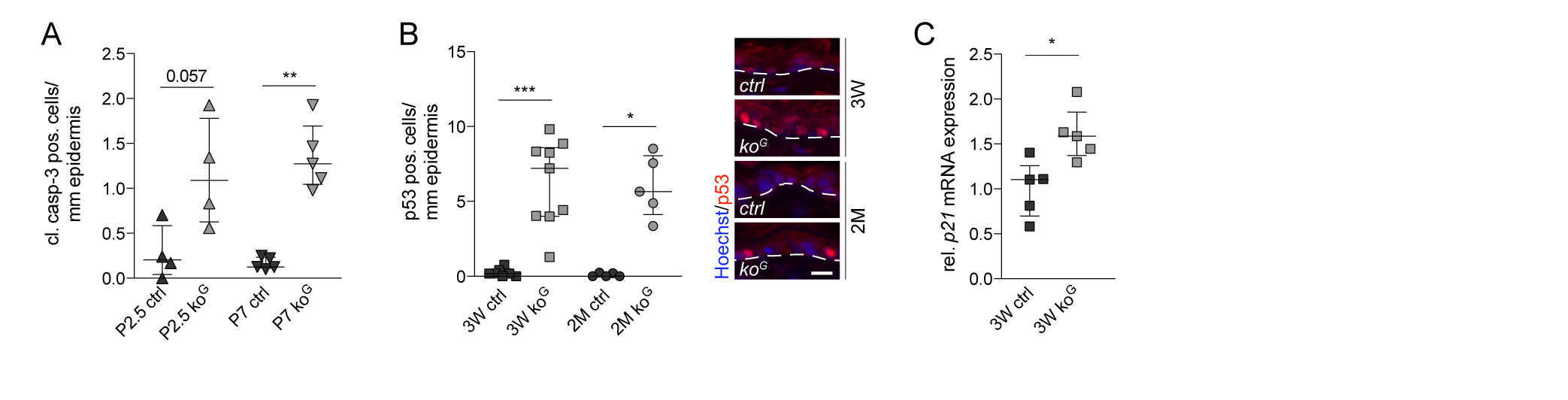

Supplement: S6 Fig — (A) Quantification of cleaved caspase-3 positive cells per length epidermis in immunofluorescence stained skin sections from control and koG mice at P2.5 and P7. P2.5: N = 4; P7: N = 5. (B) Immunofluorescence staining of skin sections at 3W (N = 8/9) and 2M (N = 5) for p53 and quantification of cells with p53-positive nuclei per length epidermis. Scale bar: 10 μm. (C) qRT-PCR analysis of p21 relative to Rps29 using RNA from the epidermis at 3W. N = 5. Scatter plots show the median with interquartile range. *P ≤ 0.05, **P ≤ 0.01, and ***P ≤ 0.001. (TIF) [file pgen.1005800.s006.tif]

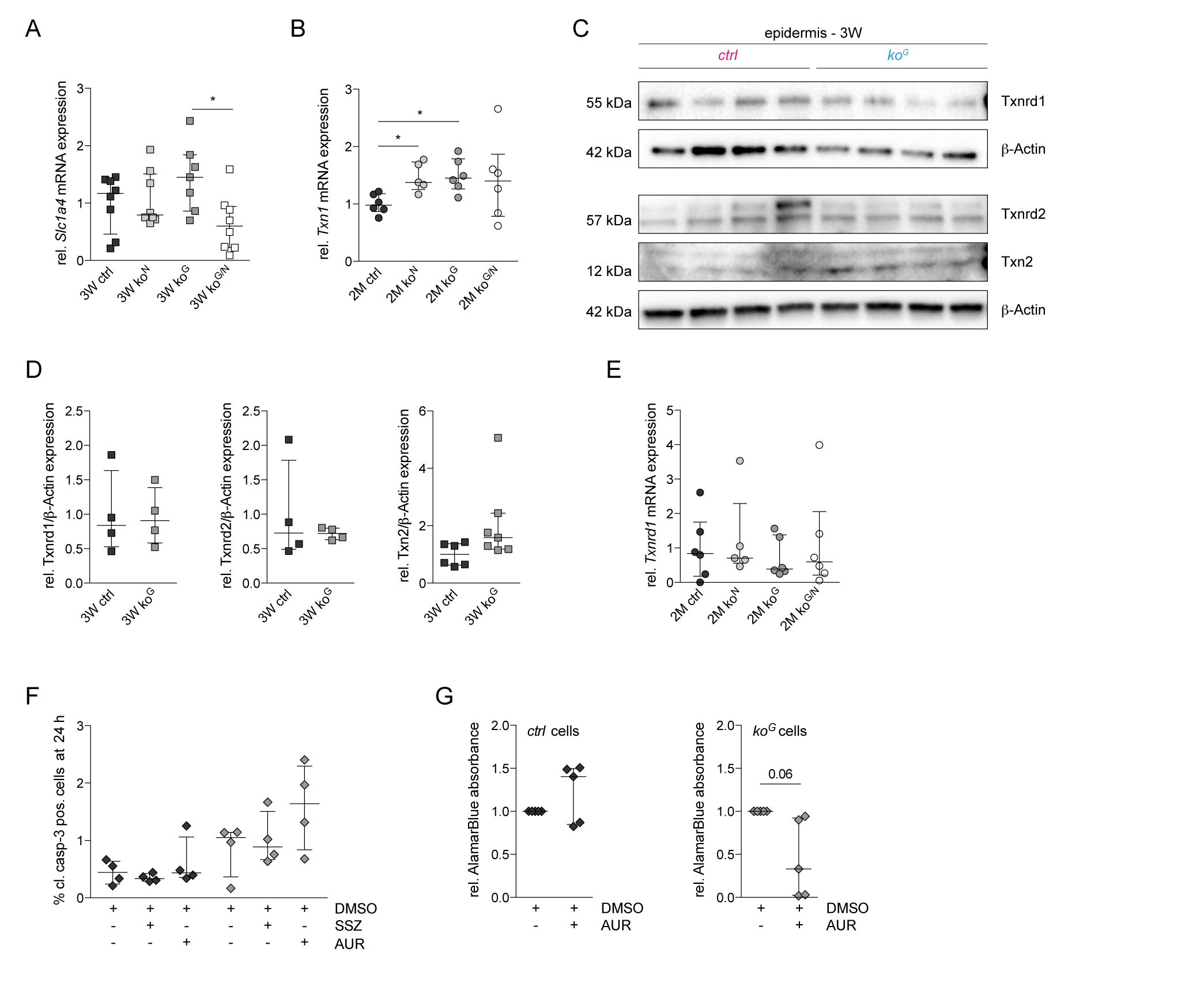

Supplement: S7 Fig — (A) qRT-PCR of Slc1a4 relative to Rps29 using RNA form the epidermis at 3W. N = 8/8/7/8. (B) qRT-PCR of Txn1 relative to Rps29 using RNA form the epidermis at 2M. N = 6/5/6/6. (C) Representative Western blot of lysates from mouse epidermis at 3W for Txnrd1, Txnrd2, Txn2 and β-actin (loading control). (D) Quantification of protein expression levels. N = 4 for Txnrd1 and Txnrd2; N = 6 for Txn2. (E) qRT-PCR of Txnrd1 relative to Rps29 using RNA form the epidermis at 2M. N = 6/5/6/6. (F) Quantification of percentage of cleaved caspase-3 positive cells in primary keratinocyte cultures from individual mice after 24 h incubation with DMSO (vehicle), SSZ or AUR (both in DMSO). N = 4. (G) AlamarBlue cell viability assay after treatment of primary keratinocytes with DMSO (vehicle) or AUR in DMSO for 24 h. N = 5, analyzed in two independent experiments. Scatter plots show the median with interquartile range. *P ≤ 0.05. (TIF) [file pgen.1005800.s007.tif]
