# Supplementary material for: A Glutathione-Nrf2-Thioredoxin Cross-Talk Ensures Keratinocyte Survival and Efficient Wound Repair
Source: PLoS Genet. 2016 Jan 25;12(1):e1005800. doi: 10.1371/journal.pgen.1005800 (PMC4726503; doi:10.1371/journal.pgen.1005800)
Supplement: S1 Table — The sequences of all forward and reverse primers used for qRT-PCR are shown. (PDF) [file pgen.1005800.s008.pdf]

Table S1: Primers used for qRT-PCR

Gene	*forward (5')*	*reverse (3')*
mAldh3a1	GCTGGAGAGGACTGTGTAGA	GGTCGAGTCTTGCCTGAGTT
mCat	AGTGTGCCATCTCGTCAGTG	CCAGATGAAGCAGTGGAAGG
mCbr1	TACCCGAGATGTCTGCAAGG	CCTCTGTGATGGTCTCGCTT
mCbr3	TCGACATTCAAGCTGAGGTGA	CCTGCAGACTGCTGATGTTCA
mElovl1	CAAGGGCAGACAATCCATAG	CCTCCATGTCTTCCACCACT
mElovl3	ATCCGTGTAGATGGCAAAGC	CCTTTTTGGAGGAGTACTGGG
mElovl4	CAGCCACACGAACAGGAGAT	GTTCTATCGCTGGACCTGGA
mElovl6	GGTACCAGTGCAGGAAGATCA	ACAATGGACCTGTCAGCAAA
mElovl7	GGCATGATCGTATGATGGAA	GCACACCTGCTGGCTTTATT
mFlg	GTCTTCTTCCAAACAAGGTGCAT	TTGCCAGCTTTAGCACCAGT
mGclc	AACAAGAAACATCCGGCATC	CGTAGCCTCGGTAAAATG GA
mGsta3	TACTTTGATGGCAGGGGAAG	GCACTTGCTGGAACATCAGA
mGulo	CCTTTTTGGAGGAGTACTGGG	GCAGCTTGCTTCTGCTTCTAC
mIl6	TTCTGCAAGTGCATCATCGT	CCGGAGAGGAGACTTCACAG
mLor	CACTCATCTTCCCTGGTGCT	TCCACCAGAGGTCTTTCCAC
mNqo1	CTGGCCCATTCAGAGAAGAC	GTCTGCAGCTTCCAGCTTCT
mNrf2	TCCATTCCCGAATTACAGTGTCTTA	CGCCAAAATCTGTGTTTAAGGTG
mp21	GTGATTGCGATGCGCTCATG	TCTCTTGCAGAAGACCAATC
mPtgs2	TCATACATTCCCCACGGTTT	GATGCTCTTCCGAGCTGTG
mRps29	GGTCACCAGCAGCTCTACTG	GTCCAACTTAATGAAGCCTATGTCC
mS100A8	GTGAGATGCCACACCCACTTT	GCCGTCTGAACTGGAGAAG
mSlc1a4	ACGGGGATCTTCTCTTTGGT	CAGAAACCTGTTCCCTTCCA
mSlc7a11	CAACAAAGATCGGGACTGCT	GCTGGCTGGTTTTACCTCAA
mSlpi	GGGCAAATACAAGTGCTGTG	CCTGGGAGCAGGGAAGTAGT

mSod1	TGCTGGCCTTCAGTTAATCC	ACCATCCACTTCGAGCAGAA
mSprr2d	CTGGTACTCAAGGCCGAGAC	CAGGGCACTTTGGTGGAG
mSrxn1	CGGTGCACAACGTACCAAT	TTGATCCAGAGGACGTCGAT
mTnfα	CCACTTGGTGGTTTGCTACGA	GACCCTCACACTCAGATCATCTTCT
mTslp	TCTGGAGATTGCATGAAGGA	CCAGGCTACCCTGAAACTGA
mTxn1	GCTTGTCGTGGTGGACTTCT	CCCCCACCTTTTGACCCTTT
mTxnrd1	GACACTCTACTAAGTGCCCTGC	GGAAACCAGCAACAGTTGGG
