# Supplementary material for: A Glutathione-Nrf2-Thioredoxin Cross-Talk Ensures Keratinocyte Survival and Efficient Wound Repair
Source: PLoS Genet. 2016 Jan 25;12(1):e1005800. doi: 10.1371/journal.pgen.1005800 (PMC4726503; doi:10.1371/journal.pgen.1005800)
Supplement: S2 Table — The antibodies used for western blotting are shown in the Table, including the antigen, the host in which the antibody was generated, the catalogue number, and the source. (PDF) [file pgen.1005800.s009.pdf]

Table S2: List of antibodies used for western blotting

Antigen	*Host*	*Cat. No.*	*Source*
β-Actin	Mouse	A5441	Sigma, Buchs, Switzerland
α-Tubulin	Mouse	T5168	Sigma, Buchs, Switzerland
Gclc	Rabbit	sc22755	Santa Cruz, Santa Cruz, CA
Filaggrin	Rabbit	PRB-417P	Biolegend, San Diego, CA
Gapdh	Mouse	5G4	HyTest, Turku, Finland
Glrx2	Rabbit	–	Dr. Carsten Berndt, Heinrich-Heine University of Düsseldorf, Germany
Gpx4	Rabbit	ab125066	Abcam, Cambridge, UK
Loricrin	Rabbit	PRB-145P	Biolegend, San Diego, CA
Prdx6	Rabbit	ab59543	Abcam, Cambridge, UK
Txn2	Goat	AF3254	R&D Systems, Minneapolis, MN
Txnrd1	Rat	–	Dr. Elisabeth Kremmer, Helmholtz Center Munich, Germany
Txnrd2	Rat	–	Dr. Elisabeth Kremmer, Helmholtz Center Munich, Germany
