# Supplementary material for: A Glutathione-Nrf2-Thioredoxin Cross-Talk Ensures Keratinocyte Survival and Efficient Wound Repair
Source: PLoS Genet. 2016 Jan 25;12(1):e1005800. doi: 10.1371/journal.pgen.1005800 (PMC4726503; doi:10.1371/journal.pgen.1005800)
Supplement: S3 Table — The antibodies used for immunostaining are shown in the Table, including the antigen, the host in which the antibody was generated, the catalogue number, and the source. (PDF) [file pgen.1005800.s010.pdf]

Table S3: List of antibodies used for immunostaining

Antigen	*Host*	*Cat. No.*	*Source*
CD3	Rabbit	A0452	Dako, Baar, Switzerland
Cleaved caspase-3	Rabbit	9661	Cell Signaling, Beverly, MA
Filaggrin	Rabbit	PRB-417P	Biolegend, San Diego, CA
pan-γδ TCR-FITC	Hamster	553177	BD Pharmingen, Franklin Lakes, NJ
γH2AX-biotin (Ser139)	Mouse	16-193	Merck, Darmstadt, Germany
Keratin 6	Rabbit	PRB-169P	Biolegend, San Diego, CA
Keratin 10	Mouse	M7002	Dako, Baar Switzerlannd
Keratin 14	Rabbit	PRB-155P	BAbCO, Richmond, CA
Ki-67	Rabbit	ab16667	Abcam, Cambridge, UK
Ki-67	Rat	M724901	Dako, Baar, Switzerlannd
Loricrin	Rabbit	PRB-145P	Biolegend, San Diego, CA
p53	Rabbit	NCL-p53-CM5p	Leica Biosystems, Wetzlar, Germany
