# Supplementary material for: A Glutathione-Nrf2-Thioredoxin Cross-Talk Ensures Keratinocyte Survival and Efficient Wound Repair
Source: PLoS Genet. 2016 Jan 25;12(1):e1005800. doi: 10.1371/journal.pgen.1005800 (PMC4726503; doi:10.1371/journal.pgen.1005800)
Supplement: S4 Table — The dyes and antibodies used for flow cytometry are shown in the Table, including the antigen/dye, the fluorophore coupled to the dye/antibody, the catalogue number, and the source. (PDF) [file pgen.1005800.s011.pdf]

Table S4: List of dyes and antibodies used for flow cytomerty

Antigen/dye	*Fluorophore*	*Cat. No.*	*Source*
Zombie violet	–	423114	Biolegend, San Diego, CA
CD45	AF700	103128	Biolegend, San Diego, CA
CD3	PE-CF594	562286	BD Biosciences, San Jose, CA
pan $\gamma\delta$ TCR	FITC	553177	BD Biosciences, San Jose, CA
CD69	APC	560689	BD Biosciences, San Jose, CA
F4/80	AF647	MCA497A647	AbD Serotec, Puchheim, Germany
Ly6G	FITC	551460	BD Biosciences, San Jose, CA
